# Supplementary material for: Value of stakeholder engagement in improving newborn care in Kenya: a qualitative description of perspectives and lessons learned
Source: BMJ Open. 2021 Jun 30;11(6):e045123. doi: 10.1136/bmjopen-2020-045123 (PMC8246352; doi:10.1136/bmjopen-2020-045123)
Supplement: Author's manuscript [file bmjopen-2020-045123.draft_revisions.pdf]

BMJ Open is committed to open peer review. As part of this commitment we make the peer review history of every article we publish publicly available.

When an article is published we post the peer reviewers' comments and the authors' responses online. We also post the versions of the paper that were used during peer review. These are the versions that the peer review comments apply to.

The versions of the paper that follow are the versions that were submitted during the peer review process. They are not the versions of record or the final published versions. They should not be cited or distributed as the published version of this manuscript.

BMJ Open is an open access journal and the full, final, typeset and author-corrected version of record of the manuscript is available on our site with no access controls, subscription charges or pay-per-view fees (http://bmjopen.bmj.com).

If you have any questions on BMJ Open's open peer review process please email info.bmjopen@bmj.com

BMJ Open

Stakeholder Engagement in improving Newborn Care in Kenya: Description, perspectives and lessons learned

Journal:	*BMJ Open*
Manuscript ID	bmjopen-2020-045123
Article Type:	Original research
Date Submitted by the Author:	23-Sep-2020
Complete List of Authors:	Nzinga, Jacinta; KEMRI-Wellcome Trust Research Programme Nairobi, Health Services Unit<br>Jones, Caroline; KEMRI-Wellcome Trust Research Programme Nairobi, Health Systems Research and Ethics; Oxford University, Nuffield Department of Medicine<br>Gathara, David; KEMRI-Wellcome Trust Research Programme Nairobi, Health Systems Research and Ethics<br>English, Mike; KEMRI-Wellcome Trust Research Programme Nairobi, Health Systems Research and Ethics ; Oxford University, Nuffield Department of Medicine
Keywords:	Organisation of health services < HEALTH SERVICES ADMINISTRATION & MANAGEMENT, Health policy < HEALTH SERVICES ADMINISTRATION & MANAGEMENT, QUALITATIVE RESEARCH

SCHOLARONE™
Manuscripts

BMJ

I, the Submitting Author has the right to grant and does grant on behalf of all authors of the Work (as defined in the below author licence), an exclusive licence and/or a non-exclusive licence for contributions from authors who are: i) UK Crown employees; ii) where BMJ has agreed a CC-BY licence shall apply, and/or iii) in accordance with the terms applicable for US Federal Government officers or employees acting as part of their official duties; on a worldwide, perpetual, irrevocable, royalty-free basis to BMJ Publishing Group Ltd ("BMJ") its licensees and where the relevant Journal is co-owned by BMJ to the co-owners of the Journal, to publish the Work in this journal and any other BMJ products and to exploit all rights, as set out in our licence.

The Submitting Author accepts and understands that any supply made under these terms is made by BMJ to the Submitting Author unless you are acting as an employee on behalf of your employer or a postgraduate student of an affiliated institution which is paying any applicable article publishing charge ("APC") for Open Access articles. Where the Submitting Author wishes to make the Work available on an Open Access basis (and intends to pay the relevant APC), the terms of reuse of such Open Access shall be governed by a Creative Commons licence – details of these licences and which Creative Commons licence will apply to this Work are set out in our licence referred to above.

Other than as permitted in any relevant BMJ Author's Self Archiving Policies, I confirm this Work has not been accepted for publication elsewhere, is not being considered for publication elsewhere and does not duplicate material already published. I confirm all authors consent to publication of this Work and authorise the granting of this licence.

For peer review only

Stakeholder Engagement in improving Newborn Care in Kenya: Description, perspectives and lessons learned

Jacinta Nzinga, 1

Caroline Jones, 1, 2

David Gathara, 1

Mike English, 1, 2

Corresponding author: Jacinta Nzinga, KEMRI-Wellcome Trust Research programme, Kenya, PO Box 43640-00100, Nairobi, Kenya. Email: jnzinga@kemri-wellcome.org

1. KEMRI-Wellcome Trust, Nairobi & Kilifi, Kenya
2. Nuffield Department of Medicine, University of Oxford, UK

Key words: stakeholders, participatory research, engagement, health systems,

Word Count

Abstract 284

Main Text 4649

Abstract

Objective: It is widely recognised that embedding researchers within health systems results in more socially relevant research and more effective uptake of evidence into policy and practice but the practice of embedded health service research remains poorly understood.We set out to explore and assess the development of embedded participatory approaches to health service research by a health research team in Kenya highlighting the different ways multiple stakeholders were engaged in a neonatal research study.

Setting: Over recent years, the Health Services Unit (HSU) within the KEMRI-Wellcome Trust Research Programme (KWTRP) in Nairobi Kenya, has been working closely with organisations and technical stakeholders including, but not limited to, medical and nursing schools, frontline health workers, senior paediatricians, policy makers and county officials,

For peer review only - http://bmjopen.bmj.com/site/about/guidelines.xhtml

in developing and conducting embedded health research. The research approach focuses on prioritizing stakeholder engagement and adapted forms of participatory action research. This involves researchers embedding themselves in the contexts in which they carry out their research (mainly in county hospitals, local universities and other training institutions), creating and sustaining social networks, and collaboratively working with stakeholders to identify clinical, operational and behavioural issues related to routine service delivery, formulating and exploring research questions to bring change in practice

Participants: We purposively selected 14 relevant stakeholders spanning policy, training institutions, healthcare workers, regulatory councils and professional associations

Results: The value of embeddedness is highlighted through the description of a recently completed project, Health Services that Deliver for Newborns (HSD-N). We describe how the HSD-N research process contributed to and further strengthened a collaborative research platform and illustrating this project's role in identifying and generating ideas about how to tackle health service delivery problems

Conclusions: We conclude with a discussion about the experiences, challenges and lessons learned regarding engaging stakeholders in the co-production of research

Article Summary

Strengths and Limitation of this study

Strengths from this article include emphasis on involvement; understanding who is and should be involved, when should this engagement occur (i.e., at what points in the research process), and how this engagement should be done (i.e., what are the approaches to engagement that yield the results).

Furthermore, successful participatory processes require; openness of dialogue with a genuine empathy for others' perspectives; active listening and courtesy; early and ongoing voice and creating meaningful decision space throughout the engagement process

However, the limitations of this study include complications by a number of context and resource-based factors including; competing priorities, tension among stakeholder groups, high staff turnover and lack of commitment

There is need for more empiric work to develop and apply explanatory theories, frameworks and models to better understand how participation occurs, under what contextual settings and what is produced

For peer review only

Introduction

Recent literature has underscored the value of health policy and systems research as an intervention for systems strengthening [1]. In the last decade there has been increased demand for embedded health systems research in low and middle-income countries (LMICs), as leverage for more socially relevant and responsive research, and for more effective uptake of evidence into action/policy/practice[2, 3]. Further, implementation research has highlighted the need for context-specific research evidence as part of solutions to address the translation of knowledge into practice[4-6].However, the uptake of research findings heavily depends on the credibility of the information produced which is in turn dependent on trusted local stakeholders' expertise and their active, meaningful involvement throughout the research process [7-9].

This paper provides a brief description of our (a health research group) history of more than 15 years of engaging with stakeholders and conducting health services research in Kenyan hospitals and explores the relational and organisational processes underlying network activities; examining the spaces in which stakeholder engagement occurred over a number of years during work which focused on hospital improvement [10-12]. It then provides a critical analysis of the most recent lessons learnt through a description of a study aimed at understanding how local structural, contextual and cultural factors influenced the research-policy-practice engagement process in a recently completed health systems research project. The aim is to provide a better understanding of the requirements of embedded participation in responding to local problems.

Study background

The Health Services Unit (HSU) of the KEMRI-Wellcome Trust Research Programme started working closely with the Ministry of Health (MoH) of Kenya in 2004 developing and implementing research on facility-based care to improve child and newborn survival [13-15]. Early work focused on developing and implementing a multifaceted intervention aimed at improving paediatric inpatient care in district hospitals in Kenya [16]. Data collection included long-term participant observation and continuous reflection on the positionality of study team members embedded in the study hospitals [17, 18]. To allow engagement with stakeholders, regular evidence synthesis meetings and feedback meetings were held with the hospitals. There were bi-monthly phone calls to understand how the intervention was unfolding as well as formal and informal discussions and consultations with the stakeholders to understand their interest in the engagement. A key lesson from the project was that

For peer review only - http://bmjopen.bmj.com/site/about/guidelines.xhtml

changing practice and system hospitals required specific collaboration with partners who are usually considered the subjects of research.

Consequently, driven by the need for system wide improvement, the HSU partnered with the MoH, the Kenyan Paediatric Associated and 14 county (district) level hospitals in 2010 to create a Clinical Information Network (CIN). The network aimed to produce high-quality process and outcome data from individual admissions to paediatric wards in Kenyan hospitals and use these data to inform improvement strategies. Through collaborative working, the network has grown into a community of practice aimed at slowly changing hospital culture through sustained engagement, peer support and linking hospitals within the network [19]. The effects of the CIN platform, critically explored through formative explanation and theory of change, are documented elsewhere [20].

Through these projects, the research team began to learn from stakeholders how contexts shape service delivery, and how relationships between the research team, health managers and health workers develop and shape the delivery of the interventions over time[21, 22].However, this research process involved limited true co-production, partly because research funding provided limited support for extensive work of this kind. Furthermore, it was apparent that the practice of embedded HPSR in LMICs was, at that time, not very well defined and that trial-and-error strategies like our own were often applied.

Over time, the research group developed a more deliberate and collaborative approach that was taken forward in subsequent projects including the HSD-N (Health Services that Deliver for Newborns) project detailed below.

The HSD-N project: 2013-2018

As a research team, concerned by the high neonatal mortality in Nairobi, we held consultative meetings with the County Government of Nairobi and other key stakeholders. Together, and whilst drawing on our 10 years' research experience on quality of care[23-25] we co-developed the HSD-N (Health Services that Deliver for Newborns) project with key stakeholders. The project aimed to address the challenges influencing the delivery of essential inpatient newborn services in Nairobi County with a particular focus on nursing care, which was highlighted by all stakeholders as a neglected topic (*figure 1*).

The initial approach to conceptualising how gaps might be addressed was informed by Kenyan policy objectives, specifically the focus at national policy level on task shifting [26] and early discussions with the Nairobi City Council (NCC) in which concerns over how newborn care was delivered across the public, private and faith-based sectors were raised. In

For peer review only - http://bmjopen.bmj.com/site/about/guidelines.xhtml

light of the prevailing policy environment our research included an explicit aim to explore the potential of task shifting through the use of health care assistants (HCAs) to support nursing care as one potentially important component for improved newborn care practice in Kenyan and possibly other LMICs [27, 28].

The HSD-N project took place in three phases (*figure 1*). At the heart of this work, was a strategic approach to researching and intervening in the health system based on collaborative engagement from the outset. Building on relationships developed from previous projects we began to forge new linkages with powerful professionals, regulators, health professional bodies, private institutions and other major decisions makers in health in Kenya. As part of this effort we deliberately sought out new partners from the nursing community in Kenya with whom we had been less involved in the past. This stakeholder network was a core facilitator for truly collaborative and co-produced research.

Phase 1 (2014-2015): The existing links developed by the HSU over the years allowed an initial drafting of a list of key stakeholders likely to play a critical role in the conduct and impact of research addressing nursing service policy and practice issues [29, 30]. The list was collaboratively reviewed by the research team and initial stakeholders with more stakeholders added following certain strategic considerations. These included: the projects' core research questions; the power and interests of those who would be responsible for making decisions informed by the research; and the individuals and groups that would be affected by such decisions. Specifically, there was a deliberate effort to engage individuals and groups involved in nursing policy formulation in the country [31] mainly through stakeholder meetings as shown in Table 1. During these meetings the appropriateness and effectiveness of the research approach adopted was heavily dependent on learning from and listening to these stakeholders.

Phase 2 (2015-2017): The empirical data collection for the HSD-N project started with two distinct bodies of work: 1. Formative ethnographic research aimed at developing a preliminary understanding of nursing culture in hospitals in Nairobi and; 2. Quantitative work that explored the quality of and need for inpatient neonatal care and nursing quality of care in Nairobi City County [32][15]. Subsequently we explored how context, including barriers, enablers and the cultures of facility-based health worker teams, guide possible task-shifting strategies. We also collated experiences of mothers whose children were hospitalised in public hospital NBUs. During this empirical phase of the project, engagement activities included stakeholder engagement meetings and workshops, various trainings and hospital feedback meetings on empirical findings (Table 1).

For peer review only - http://bmjopen.bmj.com/site/about/guidelines.xhtml

Phase 3 (2017-2018): Alongside empirical data collection a series of stakeholder workshops with nursing and neonatal care experts helped define core standards for care of sick newborns in Kenyan hospitals [24, 33]. The stakeholder workshops focused on: the capacity required to provide an essential package of services for sick newborns; understanding the nursing time/skills needed for effective delivery of interventions; and, in the later stages, developing illustrative economic models to elucidate the possible consequences of these alternatives (*figure 1*). These workshops were complimented by hospital feedback meetings and various topic-specific meetings as shown in Table 1.

(Figure 1 about here)

To provide an in-depth understanding of how the HSD-N project was developed and implemented in practice, we present a chronological timeline of the research process and how the 'engagement platform' developed, identifying the key engagement activities that were influential in enabling coproduction during the lifetime of the project (*Table 1*).

Table 1*: *chronological representation of research engagement and contribution of the HSD-N project in shaping engagement and co-production of research

**YEAR**	**2014**	**2015**	**2016**	**2017**	**2018**
**ENGAGEMENT PLATFORMS**					
**Meetings**	2 meetings with representatives from the Nairobi County health management team, with the universities, KP and MoH. These meetings	1 Meeting with County Executive Member for Health Services<br><br>Stakeholder meetings introduction to the HSD-N project<br><br>Meeting on estimating the	Expert meeting on developing Neonatal Nursing Standards of Practice<br><br>Stakeholder meetings on<br><br>Estimating the Requirement for Inpatient	Nairobi Newborn Study feedback and presentation of report meeting<br><br>Feedback meeting on results on the context issues for neonatal	Healthcare assistants costing meeting<br><br>Cross-site Hospital feedback meetings on task sharing in practice<br><br>Developing nursing

For peer review only - http://bmjopen.bmj.com/site/about/guidelines.xhtml

	were held during the drafting of the proposal through to submission for funding	Requirement for Inpatient Neonatal Care and Neonatal Burden of Disease	Neonatal Care<br><br>Basic standards of quality newborn care<br><br>Results of the Nairobi newborn study on neonatal service provision	nursing task shifting<br><br>Hospital specific feedback meetings on task sharing in practice<br><br>An introduction to survey work on missed neonatal care meeting	indicators meeting<br><br>Feedback on missed care survey work meeting
**Workshops**		Checking newborn epidemiological estimates with newborn experts<br><br>Check the facilities we identified for the survey<br><br>Disseminate the facility survey findings	'Fact-check' workshop on the early facility survey findings<br><br>Expert workshop meeting on developing Neonatal Nursing Standards of Practice	Two workshops on NHCA scope of practice and training,<br><br>On hierarchical task analysis (two of these)<br><br>On nursing missed care questionnaire design	One on levels of neonatal care<br><br>One on costing.
**Interviews**		Stakeholder mapping and collecting views on task-			End of project interviews

For peer review only - http://bmjopen.bmj.com/site/about/guidelines.xhtml

		shifting with pediatric and nursing experts, academic stakeholders			with 14 stakeholders
**Training**			Hierarchical Task Analysis meeting	Missed care observational methods training	
**Hospital specific feedback meetings**		All through			
**Multi-disciplinary quarterly researcher reflective meetings**		All through			

For peer review only

Critical Analysis of the research-policy-practice engagement process in the HSD-N Project

Methods

To explore the content and consequences of the HSD-N engagement activities over the project period, we used a combination of data collection methods including: project document analysis, key informant interviews and pre-planned observation of HSD-N meetings.

Ethical considerations

Ethical approval was obtained from the Kenya Medical Research Institute Ethical Review Committee (SCC Protocol No. 3366). Written informed consent was obtained from all the participants

For peer review only - http://bmjopen.bmj.com/site/about/guidelines.xhtml

Patient and public involvement

Patients were not involved in setting the research question or the outcome measures, but key public stakeholders who were part of the HSD-N collaborative group and described in this paper were consulted in the design, conduct and dissemination of the study findings.

Document Review

We conducted a thematic analysis of HSD-N field reports, project SOPs and published manuscripts to track which stakeholder participated in what activity, and what interests they had in the different elements of the project to build a history of engagement over the project lifetime. We also reviewed stakeholder meeting reports that were co-produced with, and endorsed by, the various stakeholders during the project. This provided an understanding of areas of consensus and disagreements on proposed clinical areas and research activities as well as stakeholder's interest and feedback on the project's implementation process.

Participant observations

We used longitudinal participant observations and reflective meetings with the HSD-N qualitative research team to collect information as the programme developed. At formal stakeholders' meetings data were collected by 4 research team members observing and taking field-notes of meeting proceedings. Documentation focused on who was participating, what and how they contributed to the discussions. The longitudinal nature of data collection (attendance at a series of meetings over time – *figure 2*) allowed for documentation of changes in an individual's views over time and thematically analysed alongside the key informant interviews.

Key informant interviews

To continue building our understanding of how neonatal care is perceived within policy and practice environments we tracked the continuing purposeful engagement with stakeholders exploring the influence of stakeholder-researcher interactions. Six months before the end of the project we conducted in-depth interviews with purposively selected key informants with potential policy influence, including: The Nursing Council of Kenya (NCK), National Nursing Association of Kenya (NNAK), Kenya Pediatric Association (KPA), various nursing training schools, private organizations, and frontline workers. Although the HSD-N project was geographically Nairobi focused, many of the groups represented national level stakeholders.

The interviews focused on what drove individuals to be part of the stakeholder network, their understanding of the project, nature of involvement, how their inputs were gathered and any impact of their involvement. All interviews were conducted in English, lasted 40mins-60mins and were audio-recorded following informed consent from participants.

For peer review only - http://bmjopen.bmj.com/site/about/guidelines.xhtml

Data analysis

Data were analysed both inductively (emerging from the interview data) and deductively driven by a priori themes around the purpose and mechanisms of engagement, researcher-stakeholder relationships, and how local structural, contextual and cultural factors influenced the process of research-policy-practice engagement [34, 35]. Through critical analysis of the empirical data and reflexivity we developed a rich description of the concerns and interests of stakeholders and health workers likely to be affected by the research findings. The findings are summarised under four main themes: classification and description of stakeholders; interpreting the HSD-N engagement; barriers and facilitators of engagement and the context and nature of engagement.

Results

Over the project's lifetime, from phase 1 through phase 3, we observed 20 meetings with stakeholders from 2015 to 2018, reviewed 6 project feedback reports and conducted interviews with 14 selected stakeholders at the end of the project in 2018

Classification and description of stakeholders

Stakeholders of the HSD-N project were primarily from the public sector which provides the majority of neonatal care in Nairobi [36]. However, some stakeholders from private and non-for-profit organizations were included.

The roles of stakeholders in the HSD-N project was linked to 4 key project activities (*table 2):* i) study planning (includes co-design of the research questions; ii) study design procedures and development of study tools); iii) study implementation (as study participants, development of modelling scenarios or training curricula, and drafting nursing standards) and iv) interpretation and translation (ambassadors of implementation and change). A participant describes her role in HSD-N as:

For peer review only

> *"R: This one [HSD-N] was different thing …. in the initial phases of the design of the project we were involved as part of the team that we were actually designing the tools and refining them and even having consensus. So, this was good… because I participated more."*
>
> *"I collected some data, they involved me in data collection on task sharing and I felt well… I felt engaged, like I can actually give people who are here, who work in Kenyatta and get their views"*

To fully understand who should be engaged, when should this engagement occur (i.e., at what points in the research process), we explored the nature of the various engagements and present in Table 2

For peer review only - http://bmjopen.bmj.com/site/about/guidelines.xhtml

Table 2: Description and roles of HSDN stakeholders

STAKEHOLDER CATEGORIES	Policy maker	Regulator	Professional association	Training institutions	Health managers	Health professional	Researchers
	Department of monitoring and evaluation<br>Department of Nursing services, Ministry of Health (MOH)<br>World Health Organization (WHO)<br>United Nations International Children's Fund (UNICEF)	Nursing council of Kenya (NCK)	Kenya Paediatric Association (KPA)<br><br>The National Nursing Association of Kenya (NNAK)	Kenya Medical Training College (KMTC)<br><br>University of Nairobi (UON)<br><br>AgaKhan University Hospital (AKUH)<br><br>Kenyatta University (KU)	Ward and departmental managers of;<br><br>Public hospitals<br>Mission hospitals<br>Private hospitals	Nurses, medical officers and clinical officers of;<br><br>Public hospitals<br>Mission hospitals<br>Private hospitals	Multi-disciplinary team of researchers from;<br><br>(Kenya Medical Research Institute-Wellcome Trust Research Programme (KEMRI-WTRP), AgaKhan University Hospital (AKUH), Strathmore University<br>Oxford University<br>Warwick University
**NATURE OF ENGAGEMENT**							

For peer review only - http://bmjopen.bmj.com/site/about/guidelines.xhtml

**Consultative**	Collaborated with the team in study design, implementation Advised on the political and regulatory landscape	Collaborated with the team by offering advice on study implementation. Advised on the political and regulatory landscape	Advised on the political and regulatory landscape	Provided technical theoretical and practical advice during various sessions of evidence generation<br><br>Major voice in design of neonatal health care assistants (NHCA) scope of work and preliminary curriculum plus potential salary	Provided technical advice during various sessions of evidence generation<br><br>Significant voice in shaping NHCA roles (some were already using helpers informally or in private sector more formally) and also suggestions on the political presentation of the NHCA cadre<br><br>Useful reflections on the practical realities in routine service provision	Provided technical advice during various sessions of evidence generation and reflective of the practical realities in routine service provision	
**Involved**			Involved in aspects of study implementation, including data collection			Involved in aspects of study implementation, including data collection	Mainly involved in evidence generation, incorporating the technical advice of

For peer review only - http://bmjopen.bmj.com/site/about/guidelines.xhtml

			Offered expert critique and suggestions on improving emerging findings (e.g. neonatal burden estimation)				various stakeholders in the analysis<br><br>Collating the interpretation of findings and implications on policy and practice
**Interpretation and translation**							
**Strategic endorsement**	Added credibility to the research evidence and enabled other big players to be part of the deliberations (e.g. NNAK, NCK)<br><br>Statutory agreement of translating study findings into	Added credibility to the research evidence and enabled other big players to be part of the deliberations (e.g. NNAK, NCK)<br><br>Offered reflections on feasibility of	Acted as ambassadors of change and implementation of study findings				

For peer review only - http://bmjopen.bmj.com/site/about/guidelines.xhtml

	policy recommendations	translating evidence into practice					

For peer review only

For peer review only - http://bmjopen.bmj.com/site/about/guidelines.xhtml

Context and nature of engagement processes

In table 1 above, we provided a categorization of stakeholders, the nature of engagement and stakeholders' perceived roles in the project over the 4-year implementation period.

We also sought stakeholder's opinions as to why they think they were invited to be part of this project and why they continued engaging with the project activities. Most participants reported they believed they had important contributions to make and that the project allowed an avenue for this while others joined out of personal interest:

> *"R: Personally, I love something that is at times, out of what I do every day… like a research I can help in boosting, …. I can change in the unit…I love doing different things from the norm that is why I felt I can be part of this. This project is beyond relevant… because our unit is…. we handle 200 babies and it is like 50% will go 50% will die. You know if are in such a project …you can do something about the situation… well I believe it is very relevant."*

As mentioned above, the HSDN project ran several activities as part of stakeholder engagement using concept mapping and focus groups, and all these activities were documented and archived to inform the process and success of the project. (*refer to Table 1 for type and purpose of meeting*). Stakeholders described these meetings as useful 'engagement spaces' that provided opportunity to not only discuss various aspects of the research but to also get updates regarding the project and included learning opportunities.

> *"R: Well, there is always the person part of it [HSDN] that you interact with people because quite often when we are working, everybody is just too busy to interact with each other"*

Particularly valued was provision of regular feedback, ensuring that the most knowledgeable stakeholders in the subject matter were present and that their views were sought and incorporated into the final reports. Feedback meetings allowed researchers to check understanding and modify interpretations and key messages. In particular, efforts by the research team to understand why there may be support or resistance to some of the potential recommendations was also important:

For peer review only - http://bmjopen.bmj.com/site/about/guidelines.xhtml

"R: In the meetings there are those people who participated in the research projects and also in the meetings, so it gave the project authority. and it made sense to the people who participated. When we hear that those who participated are also here, we also appreciate that report and the feedback and the evidence that is being presented."

"I think was a very exciting journey because we were able to share with each other, with the paediatric association, to discuss with the paediatricians and even have the consensus of where we need to be. I also I think the other exciting journey came in when I was involved as part of the cohort to do the publication."

However, during these meetings it was not always easy managing differing views and reactions regarding emerging recommendations, and it was particularly challenging dealing with the varied power dynamics from different groups and individuals. However, we

"R: If they are not listening then you still continue shouting there is no other language but of course occasionally you have to sit down think of another strategy. In such a situation that is the time when you think of who else has a voice, you have to think of who else could be having the same mind as mine so that you put the two voices together and we see whether we can be heard that is one strategy."

observed stakeholders' free and frank exchanges in voicing opinions, open disagreement and on occasions the research team taking on arbitration roles to ensure all voices were heard. During interviews, stakeholders recounted the various strategies they drew on in making sure they were heard and in respectfully disagreeing with opinions. As illustrated below, these included tapping into one's intrinsic personality, drawing support from members with similar views and using the research team as a mediator during debates

The nature of engagement that emerged was mainly both consultative and collaborative which enabled the cumulation of understanding and development of meaningful relationships.

For peer review only - http://bmjopen.bmj.com/site/about/guidelines.xhtml

Interpreting the HSD-N engagement

We were interested in the stakeholders' articulation of how research findings were established and their influence over such findings as this would potentially benefit effective implementation.

During the interviews, we reflected with stakeholders about; i) their technical capacity and ability to engage with the varied research topics ii) how their feedback was incorporated into the project and iii) ability to implement lessons from the project. Examples are provided below;

On ability to conceptually engage with the research, with experiential understanding of the research problem, stakeholder reported the importance of having technical capacity to engage;

> *"I also participated in the review of the procedure manual so I knew the procedures and when you tell me that a nurse assistant will be able to give fluids or to do blood transfusion then am going back to the rationale of that procedure. So those are some of the areas that I felt that I was actually up to the speed… having done it and having participated in the procedure development and having a rationale"*
>
> *"R: The other lesson is that indeed research works. Many of us are not exposed to doing the research but I have learnt research really does miracles because you are able to get into a challenge, deeply analyse it and by the end of the day you are able to know how best can you improve or what do you need to change so that you improve on this*

Stakeholders also felt that their feedback influenced the research process as represented below;

> *"Just the voice, convincing people that it is worth taking it up, and the fact that I am a trainer… I understand all curriculum and I understand the needs in the service delivery units I think with that in mind it [engagement] has enabled me to work with whoever towards achieving the goals of the project."*

For peer review only - http://bmjopen.bmj.com/site/about/guidelines.xhtml

Furthermore, stakeholders who had the ability, described application of new clinical information in their hospitals as reported below;

> *"R: Every time we came out of the meetings we would also come and improve things within the facility because even if we are few every time, we have meetings within our various hospitals we would bring some of the issues that we have noted within our CMEs (continuous medical assessments). So, there is already been a positive feedback and in fact use of the learning that we have done within the facilities."*
>
> *"R: In terms of impact I have learnt that there are things that you can do in a planned way and the results are better than the routine things that we do."*

According to the stakeholders, the process of cultivating long term researcher-stakeholder relationships meant respecting each other's time and commitment, continuously reviving interest in the project and clearly communicating and negotiating expectations.

> *"R: That [stakeholder engagement] kind of interaction has been quite good. Quite often when the team sent out mail, some of us try to say okay 'I have been sent this and I think I need to meet my obligation'. And of course, the person who sent is really waiting for feedback to be able to continue to go forward on whatever you have been asked to make comments on. That communication I think it has been quite good. And top of that, it hasn't been overwhelming because for this project we have been given adequate time to be able to address things and of course most of those documents they have been sending have not been these huge heavy documents that bog one down"*

Barriers and facilitators of the HSD-N engagement process

We learned to be sensitive to stakeholders' time commitments as this was perceived as highly important for continued engagement. Understanding how stakeholder integrate on-going research activities into daily work enabled bringing together people from various levels of the health sector building multi-layered perspectives of the research project in terms of its implementation

For peer review only - http://bmjopen.bmj.com/site/about/guidelines.xhtml

"R: The meetings were fairly regular and fairly spaced …so would have like once in six months, so I think the regularity was good because most people are really pressed on time"

"R: I realized we are meeting with a variety of stakeholders, from different facilities, that is terms of the levels public, private and then we have lecturers, we have doctors and the Nursing Council. I think it's a good way because they are able to listen to us the people on the lower level. What we are going through. And even as you bring out the project's last results, they were able really to compare and see actually this is something that will work."

As a research team, we learned that successful stakeholder engagement required early involvement in project design, providing pre-readings to enable informed discussion, creatively using "icebreakers," especially when engaging stakeholders with differing experiences/perspectives and clearly communicating the anticipated commitment of time and level of engagement.

"R: The study reports are available for most of us… we are able to go through the whole process of the study we are able to go through and it is available, so I think that is also a strong area for the study group."

Despite the positive feedback, the engagement over time also had some limitations. The most commonly reported barriers included competing priorities by most of the stakeholders and therefore a struggle to find time for the meetings but also, perhaps paradoxically, limited time allocated for deliberations during the stakeholder meetings.

For peer review only - http://bmjopen.bmj.com/site/about/guidelines.xhtml

R: I can say time...time factor has been... cause most of the time am not usually released from here [hospital x] I try to create my own time, so if you say like am here for the whole day, that means I have to squeeze in 2 shifts, because I usually report here at around 7:30am to 5:30pm so those are 2 shifts, I need to get 2 people to cover my shift but I really don't mind...I really don't mind."

"R: Yes, you know sometimes we just want to go to another place.

M: That is not our office?

R: Exactly, if we can be able to see how resources can be able to work for a two day out of the town. So, my issue is I never even participate fully...I am always called to work, so I have to keep rushing. So, I thought at sometimes that if allowable we could actually get out of your offices and we work even though it is one day we actually work until whatever time even if it is midnight. That way I feel it would be more relaxed. I felt that it was a bit tensed and like we need to make this decision, and this is the period we have, and we have to hurry up. I was okay with that speed, but I think at some level maybe we were leaving some other people dragging behind, so could we allocate a bit of time and also out of town."

Finally, sometimes the difficulty in finding the appropriate representation of stakeholders that the project sought to engage was a challenge. In other instances, the problem was the issue of sending a different representative of a group or organization to the meetings each time. Often new people struggled to understand the project's background, progress and future aims. Similarly, poor representation of administrative/managerial groups especially from the county which has high staff turnover diminished interest, commitment and ability to follow research activities was perceived by stakeholders as a threat to utility and sustainability:

For peer review only - http://bmjopen.bmj.com/site/about/guidelines.xhtml

> *"R: The things that were less exciting is that the administration aspect of the project involvement was missing. When I noted that the in charges of the unit or the hospitals were missing in this study, to me I felt your likelihood of sustainability of the good things you have done is questionable and likely to have a challenge. ...because there was no commitment from the administration."*

Lessons and implications

Our findings highlight the importance of purposefully selecting stakeholders to fit project needs. Clearly defining roles and expectations for both researchers and the stakeholders, and providing continuous feedback appeared key drivers of meaningful and impactful engagement[37, 38]. Perhaps more vital is mapping the dynamic nature of stakeholder's involvement over a projects' lifetime and creating opportunities to share ideas and views in 'safe' settings. We emphasize the importance of involving across-system actors who are often overlooked in such processes e.g. from frontline health workers who may help articulate and validate the research priorities and as implementors of recommendations to policy makers and regulators with the authority to formalize recommended practices.

We have shown that embedded participation requires investing in social capacity in form of openness of dialogue active listening and courtesy and respectful consideration of ideas contributed. When all elements are present, then participation processes are likely to increase involvement and legitimacy and if participants feel that their views are valued and used, this ultimately enhances how the research may be used in decision making. However, as we learned, participatory processes are complicated by a number of context and structural issues including managing divergent opinions, tensions and mistrust which require interpersonal and facilitation skills which not all academics are trained in or endowed with[39].

Furthermore, there also needs to be more reflection on how to meaningfully measure the worth of embedded participation[40, 41]. This involves including both outcome and process factors and acknowledging that participatory processes typically require long time frames to build awareness and work through existing stakeholder dynamics[42, 43]. There ought to be open discussions on how embedded engagement influences research processes; the significant risks for academics, who are required to adopt practices far from those traditionally taught and having to continuously manage group dynamics. There is need for reviewing funding structures in lieu of conflict between the emergent, dynamic yet invaluable role of engaging stakeholders in research versus strict timelines tied into specified deliverables. Lastly, the need for clearly-defined methods for evaluating participation, more

For peer review only - http://bmjopen.bmj.com/site/about/guidelines.xhtml

studies on developing and applying explanatory theories that better articulate how participation occurs within the relational contexts of coproduction.

We acknowledge: the HSD-N research team, particularly Elizabeth Kyala who helped with archiving the stakeholder engagements and the rest of the HSD-N Collaborative Group who made this work possible. We are also grateful to the health workers, and colleagues representing various stakeholder institutions who made this work possible. This paper is submitted with the approval of the Director of KEMRI.

Financial Support - This work was supported by a joint Health Systems Research Initiative grant provided by the Department for International Development, UK (DFID), Economic and Social Research Council (ESRC), Medical Research Council (MRC) and Wellcome Trust, grant number MR/M015386/1. ME is supported by a Wellcome Trust Senior Research Fellowship (#207522). The funding sources had no role in the study design, writing of the report and in the decision to submit the manuscript for publication. This paper is published with the permission of the Director of KEMRI.

Competing interests –JN, CJ, DN and ME received research grants linked to work in Kenya on topics related to this report. The authors have no financial interests to disclose

Contributorship Statement

JN conceived of the idea for the study supported by ME who obtained the funding for this project. Preparation for and conduct of the study was undertaken by JN who also undertook all the interviews, observations and the qualitative analysis with support from ME and DG. CJ provided theoretical support during analysis and write up while ME and DG contributed to the analytical interpretation of the data both in discussion with JN. JN produced the draft manuscript to which all authors contributed to its development. All authors read and approved the final manuscript.

Figure 1: Schematic of HSD-N research components, their inter-relationship and infused stakeholder engagements throughout the research cycle

References

1. WHO, *Strategy on health policy and systems research: changing the mindset*. 2012.
2. World Health Organization, *Strategy on health policy and systems research: changing the mindset*. 2012.
3. Ghaffar, A., et al., *Strengthening health systems through embedded research.* Bulletin of the World Health Organization, 2017. **95**(2): p. 87.

For peer review only - http://bmjopen.bmj.com/site/about/guidelines.xhtml

4. Peters, D.H., et al., *Implementation research: what it is and how to do it*. Bmj, 2013. **347**: p. f6753.

5. Behague, D., et al., *Evidence-based policy-making: the implications of globally-applicable research for context-specific problem-solving in developing countries*. Social Science & Medicine, 2009. **69**(10): p. 1539-1546.

6. English, M., et al., *An intervention to improve paediatric and newborn care in Kenyan district hospitals: understanding the context*. Implementation Science, 2009. **4**(1): p. 42.

7. Cairney, P. and K. Oliver, *Evidence-based policymaking is not like evidence-based medicine, so how far should you go to bridge the divide between evidence and policy?* Health research policy and systems, 2017. **15**(1): p. 1-11.

8. Langley, J., D. Wolstenholme, and J. Cooke, *'Collective making'as knowledge mobilisation: the contribution of participatory design in the co-creation of knowledge in healthcare*. BMC health services research, 2018. **18**(1): p. 585.

9. Fransman, J., *Charting a course to an emerging field of'research engagement studies': A conceptual meta-synthesis*. Research for All, 2018. **2**(2): p. 185-229.

10. Hanney, S., et al., *Engagement in research: an innovative three-stage review of the benefits for health-care performance*. Health Services and Delivery Research, 2013. **1**(8).

11. Ellen, M.E., et al., *What supports do health system organizations have in place to facilitate evidence-informed decision-making? A qualitative study*. Implementation Science, 2013. **8**(1): p. 84.

12. Boaz, A., et al., *Does the engagement of clinicians and organisations in research improve healthcare performance: a three-stage review*. BMJ open, 2015. **5**(12): p. e009415.

13. Irimu, G., et al., *Developing and introducing evidence based clinical practice guidelines for serious illness in Kenya*. Archives of disease in childhood, 2008. **93**(9): p. 799-804.

14. English, M., *Designing a theory-informed, contextually appropriate intervention strategy to improve delivery of paediatric services in Kenyan hospitals*. Implementation Science, 2013. **8**(1): p. 39.

15. Murphy, G.A., et al., *What capacity exists to provide essential inpatient care to small and sick newborns in a high mortality urban setting?-A cross-sectional study in Nairobi City County, Kenya*. PLoS One, 2018. **13**(4): p. e0196585.

16. Ayieko, P., et al., *A multifaceted intervention to implement guidelines and improve admission paediatric care in Kenyan district hospitals: a cluster randomised trial*. PLoS Med, 2011. **8**(4): p. e1001018.

For peer review only - http://bmjopen.bmj.com/site/about/guidelines.xhtml

17. Finlay, L. and B. Gough, *Reflexivity: A practical guide for researchers in health and social sciences*. 2008: John Wiley & Sons.
18. Kohl, E. and P. McCutcheon, *Kitchen table reflexivity: negotiating positionality through everyday talk*. Gender, Place & Culture, 2015. **22**(6): p. 747-763.
19. English, M., et al., *Building learning health systems to accelerate research and improve outcomes of clinical care in low-and middle-income countries*. PLoS medicine, 2016. **13**(4): p. e1001991.
20. English, M., et al., *What do we think we are doing? How might a clinical information network be promoting implementation of recommended paediatric care practices in Kenyan hospitals?* Health Research Policy and Systems, 2017. **15**(1): p. 4.
21. Kimberly, J.R. and M.J. Evanisko, *Organizational innovation: The influence of individual, organizational, and contextual factors on hospital adoption of technological and administrative innovations*. Academy of management journal, 1981. **24**(4): p. 689-713.
22. Rebchook, G.M., et al., *Bridging research and practice: Community-researcher partnerships for replicating effective interventions*. AIDS Education and Prevention, 2000. **12**: p. 49-61.
23. Aluvaala, J., et al., *Assessment of neonatal care in clinical training facilities in Kenya*. Archives of disease in childhood, 2015. **100**(1): p. 42-47.
24. Murphy, G.A.V., et al., *Expectations for nursing care in newborn units in Kenya: moving from implicit to explicit standards*. BMJ Global Health, 2018. **3**(2): p. e000645.
25. English, M., et al., *Lessons from a Health Policy and Systems Research programme exploring the quality and coverage of newborn care in Kenya*. BMJ Global Health, 2020. **5**(1).
26. Ministry of Health, *Transforming Health - Accelerating attainment of Health Goals: Kenya Health Sector Strategic Investment Plan (KHSSP) 2013 - 2017*. 2013, Government of Kenya: Nairobi.
27. Nzinga, J., et al., *Exploring the space for task shifting to support nursing on neonatal wards in Kenyan public hospitals*. Human resources for health, 2019. **17**(1): p. 18.
28. Tsiachristas, A., et al., *Effective coverage and budget implications of skill-mix change to improve neonatal nursing care: an explorative simulation study in Kenya*. BMJ global health, 2019. **4**(6).
29. Locock, L., et al., *Understanding the role of opinion leaders in improving clinical effectiveness*. Social science & medicine, 2001. **53**(6): p. 745-757.

For peer review only - http://bmjopen.bmj.com/site/about/guidelines.xhtml

30. Rycroft-Malone, J., et al., *Implementing health research through academic and clinical partnerships: a realistic evaluation of the Collaborations for Leadership in Applied Health Research and Care (CLAHRC)*. Implementation Science, 2011. **6**(1): p. 74.
31. Oluoch, D., et al., *Neonatal nursing policy and practice in Kenya: Key stakeholders and their views on task-shifting as an intervention to improve care quality*. Wellcome Open Research, 2018. **3**(35): p. 35.
32. Murphy, G., et al., *Nairobi Newborn Study: Estimating the gap between the need for and the availability, utilisation, and quality of facility-based inpatient newborn care in Nairobi, Kenya*. 2016.
33. Gathara, D., et al., *Developing metrics for nursing quality of care for low-and middle-income countries: a scoping review linked to stakeholder engagement*. Human Resources for Health, 2020. **18**: p. 1-16.
34. Fereday, J. and E. Muir-Cochrane, *Demonstrating rigor using thematic analysis: A hybrid approach of inductive and deductive coding and theme development*. International journal of qualitative methods, 2006. **5**(1): p. 80-92.
35. Gioia, D.A., K.G. Corley, and A.L. Hamilton, *Seeking qualitative rigor in inductive research: Notes on the Gioia methodology*. Organizational research methods, 2013. **16**(1): p. 15-31.
36. Murphy, G., et al., *Approach to developing stakeholder-informed and evidence-based task-shifting strategies to improve health services for sick newborns in Kenya*. 2016.
37. Guise, J.-M., et al., *A practice-based tool for engaging stakeholders in future research: a synthesis of current practices*. Journal of clinical epidemiology, 2013. **66**(6): p. 666-674.
38. Ray, K.N. and E. Miller, *Strengthening stakeholder-engaged research and research on stakeholder engagement*. Journal of comparative effectiveness research, 2017. **6**(4): p. 375-389.
39. Oliver, K., A. Kothari, and N. Mays, *The dark side of coproduction: do the costs outweigh the benefits for health research?* Health Research Policy and Systems, 2019. **17**(1): p. 33.
40. Lavallee, D.C., et al., *Stakeholder engagement in comparative effectiveness research: how will we measure success?* Journal of Comparative Effectiveness Research, 2012. **1**(5): p. 397-407.
41. Boaz, A., et al., *How to engage stakeholders in research: design principles to support improvement*. Health research policy and systems, 2018. **16**(1): p. 60.

For peer review only - http://bmjopen.bmj.com/site/about/guidelines.xhtml

42. Deverka, P.A., et al., *Stakeholder participation in comparative effectiveness research: defining a framework for effective engagement*. Journal of Comparative Effectiveness Research, 2012. **1**(2): p. 181-194.

43. Mathur, V.N., A.D. Price, and S. Austin, *Conceptualizing stakeholder engagement in the context of sustainability and its assessment*. Construction Management and Economics, 2008. **26**(6): p. 601-609.

For peer review only

For peer review only - http://bmjopen.bmj.com/site/about/guidelines.xhtml

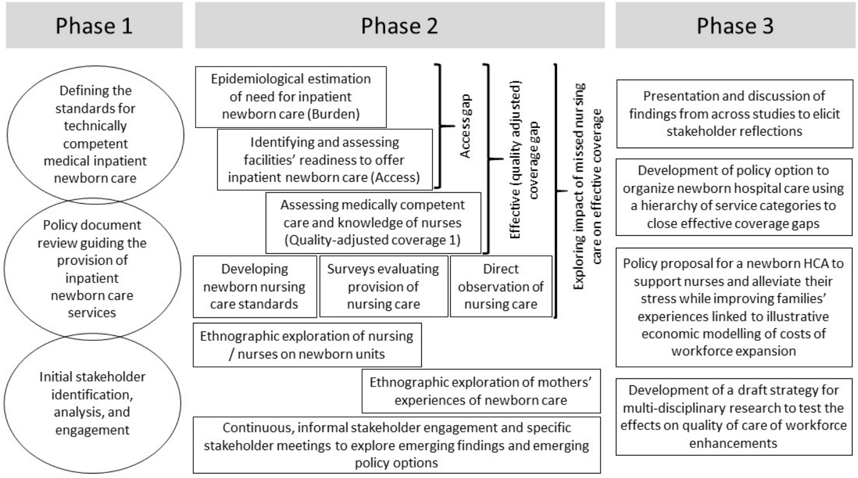

338x190mm (96 x 96 DPI)

COREQ (COnsolidated criteria for REporting Qualitative research) Checklist

A checklist of items that should be included in reports of qualitative research. You must report the page number in your manuscript where you consider each of the items listed in this checklist. If you have not included this information, either revise your manuscript accordingly before submitting or note N/A.

Topic	Item No.	Guide Questions/Description	Reported on Page No.
**Domain 1: Research team and reflexivity**			
Personal characteristics			
Interviewer/facilitator	1	Which author/s conducted the interview or focus group?	
Credentials	2	What were the researcher's credentials? E.g. PhD, MD	
Occupation	3	What was their occupation at the time of the study?	
Gender	4	Was the researcher male or female?	
Experience and training	5	What experience or training did the researcher have?	
Relationship with participants			
Relationship established	6	Was a relationship established prior to study commencement?	
Participant knowledge of the interviewer	7	What did the participants know about the researcher? e.g. personal goals, reasons for doing the research	
Interviewer characteristics	8	What characteristics were reported about the inter viewer/facilitator? e.g. Bias, assumptions, reasons and interests in the research topic	
**Domain 2: Study design**			
Theoretical framework			
Methodological orientation and Theory	9	What methodological orientation was stated to underpin the study? e.g. grounded theory, discourse analysis, ethnography, phenomenology, content analysis	
Participant selection			
Sampling	10	How were participants selected? e.g. purposive, convenience, consecutive, snowball	
Method of approach	11	How were participants approached? e.g. face-to-face, telephone, mail, email	
Sample size	12	How many participants were in the study?	
Non-participation	13	How many people refused to participate or dropped out? Reasons?	
Setting			
Setting of data collection	14	Where was the data collected? e.g. home, clinic, workplace	
Presence of non-participants	15	Was anyone else present besides the participants and researchers?	
Description of sample	16	What are the important characteristics of the sample? e.g. demographic data, date	
Data collection			
Interview guide	17	Were questions, prompts, guides provided by the authors? Was it pilot tested?	
Repeat interviews	18	Were repeat inter views carried out? If yes, how many?	
Audio/visual recording	19	Did the research use audio or visual recording to collect the data?	
Field notes	20	Were field notes made during and/or after the inter view or focus group?	
Duration	21	What was the duration of the inter views or focus group?	
Data saturation	22	Was data saturation discussed?	
Transcripts returned	23	Were transcripts returned to participants for comment and/or	

For peer review only

Topic	Item No.	Guide Questions/Description	Reported on Page No.
		correction?	
**Domain 3: analysis and findings**			
Data analysis			
Number of data coders	24	How many data coders coded the data?	
Description of the coding tree	25	Did authors provide a description of the coding tree?	
Derivation of themes	26	Were themes identified in advance or derived from the data?	
Software	27	What software, if applicable, was used to manage the data?	
Participant checking	28	Did participants provide feedback on the findings?	
Reporting			
Quotations presented	29	Were participant quotations presented to illustrate the themes/findings? Was each quotation identified? e.g. participant number	
Data and findings consistent	30	Was there consistency between the data presented and the findings?	
Clarity of major themes	31	Were major themes clearly presented in the findings?	
Clarity of minor themes	32	Is there a description of diverse cases or discussion of minor themes?	

Developed from: Tong A, Sainsbury P, Craig J. Consolidated criteria for reporting qualitative research (COREQ): a 32-item checklist for interviews and focus groups. *International Journal for Quality in Health Care*. 2007. Volume 19, Number 6: pp. 349 – 357

Once you have completed this checklist, please save a copy and upload it as part of your submission. DO NOT include this checklist as part of the main manuscript document. It must be uploaded as a separate file.

For peer review only

BMJ Open

The value of Stakeholder Engagement in improving Newborn Care in Kenya: A Qualitative Description of perspectives and lessons learned

Journal:	*BMJ Open*
Manuscript ID	bmjopen-2020-045123.R1
Article Type:	Original research
Date Submitted by the Author:	15-Mar-2021
Complete List of Authors:	Nzinga, Jacinta; KEMRI-Wellcome Trust Research Programme Nairobi, Health Services Unit<br>Jones, Caroline; KEMRI-Wellcome Trust Research Programme Nairobi, Health Systems Research and Ethics; Oxford University, Nuffield Department of Medicine<br>Gathara, David; KEMRI-Wellcome Trust Research Programme Nairobi, Health Systems Research and Ethics<br>English, Mike; KEMRI-Wellcome Trust Research Programme Nairobi, Health Systems Research and Ethics ; Oxford University, Nuffield Department of Medicine
**Primary Subject Heading**:	Health services research
Secondary Subject Heading:	Health policy, Qualitative research
Keywords:	Organisation of health services < HEALTH SERVICES ADMINISTRATION & MANAGEMENT, Health policy < HEALTH SERVICES ADMINISTRATION & MANAGEMENT, QUALITATIVE RESEARCH, NEONATOLOGY

SCHOLARONE™
Manuscripts

I, the Submitting Author has the right to grant and does grant on behalf of all authors of the Work (as defined in the below author licence), an exclusive licence and/or a non-exclusive licence for contributions from authors who are: i) UK Crown employees; ii) where BMJ has agreed a CC-BY licence shall apply, and/or iii) in accordance with the terms applicable for US Federal Government officers or employees acting as part of their official duties; on a worldwide, perpetual, irrevocable, royalty-free basis to BMJ Publishing Group Ltd ("BMJ") its licensees and where the relevant Journal is co-owned by BMJ to the co-owners of the Journal, to publish the Work in this journal and any other BMJ products and to exploit all rights, as set out in our licence.

The Submitting Author accepts and understands that any supply made under these terms is made by BMJ to the Submitting Author unless you are acting as an employee on behalf of your employer or a postgraduate student of an affiliated institution which is paying any applicable article publishing charge ("APC") for Open Access articles. Where the Submitting Author wishes to make the Work available on an Open Access basis (and intends to pay the relevant APC), the terms of reuse of such Open Access shall be governed by a Creative Commons licence – details of these licences and which Creative Commons licence will apply to this Work are set out in our licence referred to above.

Other than as permitted in any relevant BMJ Author's Self Archiving Policies, I confirm this Work has not been accepted for publication elsewhere, is not being considered for publication elsewhere and does not duplicate material already published. I confirm all authors consent to publication of this Work and authorise the granting of this licence.

For peer review only

The value of Stakeholder Engagement in improving Newborn Care in Kenya: A Qualitative Description of perspectives and lessons learned

Jacinta Nzinga, 1

Caroline Jones, 1, 2

David Gathara, 1

Mike English, 1, 2

Corresponding author: Jacinta Nzinga, KEMRI-Wellcome Trust Research programme, Kenya, PO Box 43640-00100, Nairobi, Kenya. Email: jnzinga@kemri-wellcome.org

1. KEMRI-Wellcome Trust, Nairobi & Kilifi, Kenya
2. Nuffield Department of Medicine, University of Oxford, UK

Key words: stakeholders, participatory research, engagement, health systems,

Word Count

Abstract 291

Main Text 4607

Abstract

Objective: Embedding researchers within health systems results in more socially relevant research and more effective uptake of evidence into policy and practice. However, the practice of embedded health service research remains poorly understood. We explored and assessed the development of embedded participatory approaches to health service research by a health research team in Kenya highlighting the different ways multiple stakeholders were engaged in a neonatal research study.

Methods: We conducted semi-structured qualitative interviews with key stakeholders. Data was analysed thematically using both inductive and deductive approaches.

Setting: Over recent years, the Health Services Unit (HSU) within the KEMRI-Wellcome Trust Research Programme (KWTRP) in Nairobi Kenya, has been working closely with

For peer review only - http://bmjopen.bmj.com/site/about/guidelines.xhtml

organisations and technical stakeholders including, but not limited to, medical and nursing schools, frontline health workers, senior paediatricians, policy makers and county officials, in developing and conducting embedded health research.. This involves researchers embedding themselves in the contexts in which they carry out their research (mainly in county hospitals, local universities and other training institutions), creating and sustaining social networks. Researchers collaboratively worked with stakeholders to identify clinical, operational and behavioural issues related to routine service delivery, formulating and exploring research questions to bring change in practice

Participants: We purposively selected 14 relevant stakeholders spanning policy, training institutions, healthcare workers, regulatory councils and professional associations

Results: The value of embeddedness is highlighted through the description of a recently completed project, Health Services that Deliver for Newborns (HSD-N). We describe how the HSD-N research process contributed to and further strengthened a collaborative research platform and illustrating this project's role in identifying and generating ideas about how to tackle health service delivery problems

Conclusions: We conclude with a discussion about the experiences, challenges and lessons learned regarding engaging stakeholders in the co-production of research

Article Summary

Strengths and Limitation of this study

Strengths from this article include emphasis on involvement; understanding who is and should be involved, when should this engagement occur (i.e., at what points in the research process), and how this engagement should be done (i.e., what are the approaches to engagement that yield the results).

Furthermore, successful participatory processes require; openness of dialogue with a genuine empathy for others' perspectives; active listening and courtesy; early and ongoing voice and creating meaningful decision space throughout the engagement process

However, the limitations of this study include complications by a number of context and resource-based factors including; competing priorities, tension among stakeholder groups, high staff turnover and lack of commitment

There is need for more empiric work to develop and apply explanatory theories, frameworks and models to better understand how participation occurs, under what contextual settings and what is produced

For peer review only - http://bmjopen.bmj.com/site/about/guidelines.xhtml

Introduction

Recent literature has underscored the value of health policy and systems research as an intervention for systems strengthening [1]. In the last decade there has been increased demand for embedded health systems research in low and middle-income countries (LMICs), as leverage for more socially relevant and responsive research, and for more effective uptake of evidence into action/policy/practice[2, 3]. Further, implementation research has highlighted the need for context-specific research evidence as part of solutions to address the translation of knowledge into practice[4-6].However, the uptake of research findings heavily depends on the credibility of the information produced which is in turn dependent on trusted local stakeholders' expertise and their active, meaningful involvement throughout the research process [7-9].

This paper provides a brief description of our (a health research group) history of more than 15 years of engaging with stakeholders and conducting health services research in Kenyan hospitals and explores the relational and organisational processes underlying network activities; examining the spaces in which stakeholder engagement occurred over a number of years during work which focused on hospital improvement [10-12]. It then provides a critical analysis of the most recent lessons learnt through a description of a study aimed at understanding how local structural, contextual and cultural factors influenced the research-policy-practice engagement process in a recently completed health systems research project. The aim is to provide a better understanding of the requirements of embedded participation in responding to local problems.

Study background

The Health Services Unit (HSU) of the KEMRI-Wellcome Trust Research Programme started working closely with the Ministry of Health (MoH) of Kenya in 2004 developing and implementing research on facility-based care to improve child and newborn survival [13-15]. Early work focused on developing and implementing a multifaceted intervention aimed at improving paediatric inpatient care in district hospitals in Kenya [16]. Data collection included long-term participant observation and continuous reflection on the positionality of study team members embedded in the study hospitals [17, 18]. To allow engagement with stakeholders, regular evidence synthesis meetings and feedback meetings were held with the hospitals. There were bi-monthly phone calls to understand how the intervention was unfolding as well as formal and informal discussions and consultations with the stakeholders to understand their interest in the engagement. A key lesson from the project was that

For peer review only - http://bmjopen.bmj.com/site/about/guidelines.xhtml

changing practice and system hospitals required specific collaboration with partners who are usually considered the subjects of research.

Consequently, driven by the need for system wide improvement, the HSU partnered with the MoH, the Kenyan Paediatric Association and 14 county (district) level hospitals in 2010 to create a Clinical Information Network (CIN) spread over 16 counties in eastern, western and central Kenya[19]. The network aimed to produce high-quality process and outcome data from individual admissions to paediatric wards in Kenyan hospitals and use these data to inform improvement strategies. Through collaborative working, the network has grown into a community of practice aimed at slowly changing hospital culture through sustained engagement, peer support and linking hospitals within the network [20]. The effects of the CIN platform, critically explored through formative explanation and theory of change, are documented elsewhere [21].

Through these projects, the research team began to learn from stakeholders how contexts shape service delivery, and how relationships between the research team, health managers and health workers develop and shape the delivery of the interventions over time[22, 23].However, this research process involved limited true co-production, partly because research funding provided limited support for extensive work of this kind. Furthermore, it was apparent that the practice of embedded Health Policy and Systems Research (HPSR) in LMICs was, at that time, not very well defined and that trial-and-error strategies like our own were often applied.

Over time, the research group developed a more deliberate and collaborative approach that was taken forward in subsequent projects including the HSD-N project detailed below.

The HSD-N project: 2013-2018

As a research team, concerned by the high neonatal mortality in Nairobi, we held consultative meetings with the County Government of Nairobi and other key stakeholders. Together, and whilst drawing on our 10 years' research experience on quality of care[24-26] we co-developed the HSD-N project with key stakeholders. The project aimed to address the challenges influencing the delivery of essential inpatient newborn services in Nairobi County with a particular focus on nursing care, which was highlighted by all stakeholders as a neglected topic (*figure 1*).

The initial approach to conceptualising how gaps might be addressed was informed by Kenyan policy objectives, specifically the focus at national policy level on task shifting [27] and early discussions with the Nairobi City Council (NCC) in which concerns over how

For peer review only - http://bmjopen.bmj.com/site/about/guidelines.xhtml

newborn care was delivered across the public, private and faith-based sectors were raised. In light of the prevailing policy environment our research included an explicit aim to explore the potential of task shifting through the use of health care assistants (HCAs) to support nursing care as one potentially important component for improved newborn care practice in Kenyan and possibly other LMICs [28, 29].

The HSD-N project took place in three phases (*figure 1*). At the heart of this work, was a strategic approach to researching and intervening in the health system based on collaborative engagement from the outset. Building on relationships developed from previous projects we began to forge new linkages with powerful (had authority to influence key policy decisions in newborn care) professionals including regulators, health professional bodies, private institutions and other major decisions makers in health in Kenya[30]. This stakeholder network was a core facilitator for truly collaborative and co-produced research.

Phase 1 (2014-2015): The existing links developed by the HSU over the years allowed an initial drafting of a list of key stakeholders likely to play a critical role in the conduct and impact of research addressing nursing service policy and practice issues [31, 32]. The list was collaboratively reviewed by the research team and initial stakeholders with more stakeholders added following certain strategic considerations. These included: the projects' core research questions; the power and interests of those who would be responsible for making decisions informed by the research; and the individuals and groups that would be affected by such decisions. Specifically, during stakeholder meetings, the appropriateness and effectiveness of the research approach adopted was heavily dependent on learning from and listening to these stakeholders.

Phase 2 (2015-2017): The empirical data collection for the HSD-N project started with two distinct bodies of work see Fig 1[15, 33].). During this empirical phase of the project, engagement activities included stakeholder engagement meetings and workshops, various trainings and hospital feedback meetings on empirical findings (Table 1).

Phase 3 (2017-2018): Alongside empirical data collection a series of stakeholder workshops with nursing and neonatal care experts helped define core standards for care of sick newborns in Kenyan hospitals [25, 34]. The stakeholder workshops focused on: the capacity required to provide an essential package of services for sick newborns; understanding the nursing time/skills needed for effective delivery of interventions and were complimented by hospital feedback meetings and various topic-specific meetings as shown in Table 1.

For peer review only - http://bmjopen.bmj.com/site/about/guidelines.xhtml

(Figure 1 about here) Fig 1: Schematic of HSD-N research components, their inter-relationship and infused stakeholder engagements throughout the research cycle

To provide an in-depth understanding of how the HSD-N project was developed and implemented in practice, we present a chronological timeline of the research process and how the 'engagement platform' developed, identifying the key engagement activities that were influential in enabling coproduction during the lifetime of the project (*Table 1*).

Table 1*: *chronological representation of research engagement and contribution of the HSD-N project in shaping engagement and co-production of research

**YEAR**	**2014**	**2015**	**2016**	**2017**	**2018**
**ENGAGEMENT PLATFORMS**					
**Meetings**	2 meetings with representatives from the Nairobi County health management team, with the universities, KP and MoH. These meetings were held during the drafting of the proposal through to submission for funding	1 Meeting with County Executive Member for Health Services<br><br>Stakeholder meetings introduction to the HSD-N project<br><br>Meeting on estimating the Requirement for Inpatient Neonatal Care and Neonatal Burden of Disease	Expert meeting on developing Neonatal Nursing Standards of Practice<br><br>Stakeholder meetings on<br><br>Estimating the Requirement for Inpatient Neonatal Care<br><br>Basic standards of quality newborn care<br><br>Results of the Nairobi newborn study on neonatal service provision	Nairobi Newborn Study feedback and presentation of report meeting<br><br>Feedback meeting on results on the context issues for neonatal nursing task shifting<br><br>Hospital specific feedback meetings on task sharing in practice<br><br>An introduction to survey work on missed neonatal care meeting	Healthcare assistants costing meeting<br><br>Cross-site Hospital feedback meetings on task sharing in practice<br><br>Developing nursing indicators meeting<br><br>Feedback on missed care survey work meeting
**Workshops**		Checking newborn epidemiological estimates with newborn experts	'Fact-check' workshop on the early facility survey findings	Two workshops on NHCA scope of practice and training,	One on levels of neonatal care<br><br>One on costing.

For peer review only - http://bmjopen.bmj.com/site/about/guidelines.xhtml

		Check the facilities we identified for the survey<br><br>Disseminate the facility survey findings	Expert workshop meeting on developing Neonatal Nursing Standards of Practice	On hierarchical task analysis (two of these)<br><br>On nursing missed care questionnaire design	
**Interviews**		Stakeholder mapping and collecting views on task-shifting with pediatric and nursing experts, academic stakeholders			End of project interviews with 14 stakeholders
**Training**			Hierarchical Task Analysis meeting	Missed care observational methods training	
**Hospital specific feedback meetings**		All through			
**Multi-disciplinary quarterly researcher reflective meetings**		All through			

For peer review only

Methods

Study setting

To explore the content and consequences of the HSD-N engagement activities over the project period, we conducted key informant interviews and pre-planned observation of HSD-N meetings within Nairobi County.

Ethics Approval

Ethical approval was obtained from the Kenya Medical Research Institute Ethical Review Committee (Approval number SERU 3366). Written informed consent was obtained from all the participants

For peer review only - http://bmjopen.bmj.com/site/about/guidelines.xhtml

Patient and public involvement

Patients were not involved in setting the research question or the outcome measures, but key public stakeholders who were part of the HSD-N collaborative group and described in this paper were consulted in the design, conduct and dissemination of the study findings.

Key informant interviews

To build our understanding of how neonatal care is perceived within policy and practice environments we tracked the continuing purposeful engagement with stakeholders exploring the influence of stakeholder-researcher interactions. Six months before the end of the project we conducted in-depth interviews with purposively selected key informants with potential policy influence, including: The Nursing Council of Kenya (NCK), National Nursing Association of Kenya (NNAK), Kenya Pediatric Association (KPA), various nursing training schools, private organizations, and frontline workers. Selected participants included both males and females, with varied years of working experience and with specific expertise in newborn care. Although the HSD-N project was geographically Nairobi focused, many of the groups represented national level stakeholders.

The interviews were guided by a pilot tested interview guide that focused on what drove individuals to be part of the stakeholder network, their understanding of the project, nature of involvement, how their inputs were gathered and any impact of their involvement. All interviews were conducted in English, within participants' work premises and lasted 40mins- 60mins. The interviews were audio-recorded following informed consent from participants and field notes taken during and after the interviews.

Data analysis

Data were analysed both inductively (emerging from the interview data and observation notes) and deductively driven by a priori themes and coded using Nvivo Qualitative software. Data was coded around the purpose and mechanisms of engagement, researcher-stakeholder relationships, and how local structural, contextual and cultural factors influenced the process of research-policy-practice engagement [35, 36].Through critical analysis of the empirical data and reflexivity we developed a rich description of the concerns and interests of stakeholders likely to be affected by the research findings. The findings are summarised under four main themes: classification and description of stakeholders; interpreting the HSD-N engagement; barriers and facilitators of engagement and the context and nature of engagement.

For peer review only - http://bmjopen.bmj.com/site/about/guidelines.xhtml

Results

The results we present are based on interviews with 14 selected stakeholders at the end of the HSD-N project in 2018 and presented under 4 main themes (*see Table 2*)

Table 2. Description of the emerging themes and sub-themes

	THEMES	SUB-THEMES
1.	Classification and description of stakeholders	Stakeholder identification process
		Nature of engagement
		Level of engagement
2.	Context and nature of engagement	Perceived value of stakeholder meetings
		Role of feedback in shaping engagement
		Strategies used in managing voices of the various stakeholders
3.	Interpreting the HSD-N engagement	Technical capacity to engage with various research topics
		Ability to implement lessons from research project
4.	Facilitator and barriers of the engagement	Early engagement in the project
		Creating safe spaces for deliberations
		Multi-level actor engagement
		Stakeholders' competing priorities
		Perceived 'poor' compensation
		High stakeholder turn-over

Classification and description of stakeholders

Stakeholders of the HSD-N project were primarily from the public sector which provides the majority of neonatal care in Nairobi [37]. However, some stakeholders from private and non-for-profit organizations were included. None of the stakeholders were compensated for their time on the project although there were in-built mechanisms to build capacity through short trainings on research and select relevant quality improvement topics. The roles of stakeholders in the HSD-N project was linked to 4 key project activities (*table 3):* i) study planning (includes co-design of the research questions; ii) study design procedures and development of study tools); iii) study implementation (as study participants, development

For peer review only - http://bmjopen.bmj.com/site/about/guidelines.xhtml

of modelling scenarios or training curricula, and drafting nursing standards) and iv) interpretation and translation (ambassadors of implementation and change).

> *"R: This one [HSD-N] was different thing .... in the initial phases of the design of the project we were involved as part of the team that we were actually designing the tools and refining them and even having consensus. So, this was good... because I participated more." Female senior university lecturer*
>
> *"I collected some data, they involved me in data collection on task sharing and I felt well... I felt engaged, like I can actually give people who are here, who work in Kenyatta and get their views" Female nurse manager*

To fully understand who should be engaged, when should this engagement occur (i.e., at what points in the research process), we explored the nature of the various engagements and present in Table 3

For peer review only - http://bmjopen.bmj.com/site/about/guidelines.xhtml

Table 3: Description and roles of HSDN stakeholders

STAKEHOLDER CATEGORIES	Policy maker	Regulator	Professional association	Training institutions	Health managers	Health workers	Researchers
	Department of monitoring and evaluation<br>Department of Nursing services, Ministry of Health (MOH)<br>World Health Organization (WHO)<br>United Nations International Children's Fund (UNICEF)	Nursing council of Kenya (NCK)	Kenya Paediatric Association (KPA)<br><br>The National Nursing Association of Kenya (NNAK)	Kenya Medical Training College (KMTC)<br><br>University of Nairobi (UON)<br><br>AgaKhan University Hospital (AKUH)<br><br>Kenyatta University (KU)	Ward and departmental managers of;<br><br>Public hospitals<br>Mission hospitals<br>Private hospitals	Nurses, medical officers and clinical officers of;<br><br>Public hospitals<br>Mission hospitals<br>Private hospitals	Multi-disciplinary team of researchers from;<br><br>(Kenya Medical Research Institute-Wellcome Trust Research Programme (KEMRI-WTRP), AgaKhan University Hospital (AKUH), Strathmore University<br>Oxford University<br>Warwick University
**NATURE OF ENGAGEMENT**							
**Consultative**	Collaborated with the team in study design, implementation<br>Advised on the political and regulatory landscape	Collaborated with the team by offering advice on study implementation.<br>Advised on the political and	Advised on the political and regulatory landscape	Provided technical theoretical and practical advice during various sessions of evidence generation	Provided technical advice during various sessions of evidence generation<br><br>Significant voice in shaping NHCA roles (some were already using helpers informally or in	Provided technical advice during various sessions of evidence generation and reflective of the practical realities in routine service provision	

For peer review only - http://bmjopen.bmj.com/site/about/guidelines.xhtml

		regulatory landscape		Major voice in design of neonatal health care assistants (NHCA) scope of work and preliminary curriculum plus potential salary	private sector more formally) and also suggestions on the political presentation of the NHCA cadre<br><br>Useful reflections on the practical realities in routine service provision		
**Involved**			Involved in aspects of study implementation, including data collection<br><br>Offered expert critique and suggestions on improving emerging findings (e.g. neonatal burden estimation)			Involved in aspects of study implementation, including data collection	Mainly involved in evidence generation, incorporating the technical advice of various stakeholders in the analysis<br><br>Collating the interpretation of findings and implications on policy and practice
**Interpretation and translation**							
**Strategic endorsement**	Added credibility to the research evidence and enabled other big	Added credibility to the research evidence and	Acted as ambassadors of change and				

For peer review only

	players to be part of the deliberations (e.g. NNAK, NCK)<br><br>Statutory agreement of translating study findings into policy recommendations	enabled other big players to be part of the deliberations (e.g. NNAK, NCK)<br><br>Offered reflections on feasibility of translating evidence into practice	implementation of study findings				

For peer review only

For peer review only - http://bmjopen.bmj.com/site/about/guidelines.xhtml

Context and nature of engagement processes

In table 3 above, we provided a categorization of stakeholders, the nature of engagement and stakeholders' perceived roles in the project over the 4-year implementation period.

We also sought stakeholder's opinions as to why they think they were invited to be part of this project and why they continued engaging with the project activities. Most participants reported they believed they had important contributions to make and that the project allowed an avenue for this while others joined out of personal interest:

> *"R: Personally, I love something that is out of what I do every day... like research can help in boosting, ... I can change in the unit...I love doing different things from the norm that is why I felt I can be part of this. This project is beyond relevant... because our unit is.... we handle 200 babies and it is like 50% will go 50% will die. You know if are in such a project ...you can do something about the situation... well I believe it is very relevant."* Male paediatrician
>
> *"R: Well, there is always the person part of it [HSDN] that you interact with people because quite often when we are working, everybody is just too busy to interact with each other"* Female paediatrician

As mentioned above, the HSDN project ran several activities as part of stakeholder engagement using concept mapping and focus groups, and all these activities were documented and archived to inform the process and success of the project. (*refer to Table 1 for type and purpose of meeting*). Stakeholders described these meetings as useful 'engagement spaces' that provided opportunity to not only discuss various aspects of the research but to also get updates regarding the project and included learning opportunities.

Particularly valued was provision of regular feedback, ensuring that the most knowledgeable stakeholders in the subject matter were present and that their views were sought and incorporated into the final reports. Feedback meetings allowed researchers to check understanding and modify interpretations and key messages. In particular, efforts by the research team to understand why there may be support or resistance to some of the potential recommendations was also important.

For peer review only - http://bmjopen.bmj.com/site/about/guidelines.xhtml

However, during these meetings it was not always easy managing differing views and reactions regarding emerging recommendations, and it was particularly challenging dealing with the varied power dynamics from different groups and individuals. However, we observed stakeholders' free and frank exchanges in voicing opinions, open disagreement and on occasions the research team taking on arbitration roles to ensure all voices were heard. During interviews, stakeholders recounted the various strategies they drew on in making sure they were heard and in respectfully disagreeing with opinions as illustrated below, The nature of engagement that emerged was mainly both consultative and collaborative which enabled the cumulation of understanding and development of meaningful relationships.

Interpreting the HSD-N engagement

We were interested in the stakeholders' articulation of how research findings were established and their influence over such findings as this would potentially benefit effective implementation.

"R: In the meetings there are those people who participated in the research projects and also in the meetings, so it gave the project authority. and it made sense to the people who participated. When we hear that those who participated are also here, we also appreciate that report and the feedback and the evidence that is being presented." Male, Professional association

"I think was a very exciting journey because we were able to share with each other, with the paediatric association, to discuss with the paediatricians and even have the consensus of where we need to be. I also I think the other exciting journey came in when I was involved as part of the cohort to do the publication." Female, Regulatory Council

"R: If they are not listening then you still continue shouting there is no other language but of course occasionally you have to sit down think of another strategy. In such a situation that is the time when you think of who else has a voice, you have to think of who else could be having the same mind as mine so that you put the two voices together and we see whether we can be heard that is one strategy." Female frontline nurse

For peer review only - http://bmjopen.bmj.com/site/about/guidelines.xhtml

During the interviews, we reflected with stakeholders about; i) their technical capacity and ability to engage with the varied research topics ii) how their feedback was incorporated into the project and iii) ability to implement lessons from the project. Examples are provided below;

On ability to conceptually engage with the research, with experiential understanding of the research problem, stakeholder reported the importance of having technical capacity to engage and also felt that their feedback influenced the research process Furthermore, stakeholders who had the ability, described application of new clinical information in their hospitals

> *"I also participated in the review of the procedure manual so I knew the procedures and when you tell me that a nurse assistant will be able to give fluids or to do blood transfusion then am going back to the rationale of that procedure"* Female nurse manager
>
> *"Just the voice, convincing people that it is worth taking it up, and the fact that I am a trainer… I understand all curriculum and I understand the needs in the service delivery units I think with that in mind it [engagement] has enabled me to work with whoever towards achieving the goals of the project.*" Female lecturer, training college
>
> *"R: Every time we came out of the meetings we would also come and improve things within the facility. So, there is already been a positive feedback and in fact use of the learning that we have done within the facilities."* Female Paediatrician

According to the stakeholders, the process of cultivating long term researcher-stakeholder relationships meant respecting each other's time and commitment, continuously reviving interest in the project and clearly communicating and negotiating expectations.

Barriers and facilitators of the HSD-N engagement process

We learned to be sensitive to stakeholders' time commitments as this was perceived as highly important for continued engagement. Understanding how stakeholder integrate on-going research activities into daily work enabled bringing together people from various levels of the

health sector building multi-layered perspectives of the research project in terms of its implementation

As a research team, we learned that successful stakeholder engagement required early involvement in project design, providing pre-readings to enable informed discussion, creatively using "icebreakers," especially when engaging stakeholders with differing experiences/perspectives and clearly communicating the anticipated commitment of time and level of engagement.

> *"R: That [stakeholder engagement] kind of interaction has been quite good. Quite often when the team sent out mail, some of us try to say okay 'I have been sent this and I think I need to meet my obligation'. That communication I think it has been quite good. And top of that, it hasn't been overwhelming because for this project we have been given adequate time to be able to address things and of course most of those documents they have been sending have not been these huge heavy documents that bog one down"* Female lecturer, training institution

> *"R: The meetings were fairly regular and fairly spaced …so would have like once in six months, so I think the regularity was good because most people are really pressed on time"* Female, frontline nurse
>
> *"R: I realized we are meeting with a variety of stakeholders, from different facilities, that is terms of the levels public, private and then we have lecturers, we have doctors and the Nursing Council. I think it's a good way because they are able to listen to us the people on the lower level. What we are going through…, they were able really to compare and see actually this is something that will work."* Female, Professional Association
>
> *"R: The study reports are available for most of us… we are able to go through the whole process of the study we are able to go through and it is available, so I think that is also a strong area for the study group."* Male, training institution

For peer review only - http://bmjopen.bmj.com/site/about/guidelines.xhtml

1

For peer review only

For peer review only - http://bmjopen.bmj.com/site/about/guidelines.xhtml

Despite the positive feedback, the engagement over time also had some limitations. The most

For peer review only

For peer review only - http://bmjopen.bmj.com/site/about/guidelines.xhtml

1 commonly reported barriers included competing priorities by most of the stakeholders and

For peer review only

For peer review only - http://bmjopen.bmj.com/site/about/guidelines.xhtml

therefore a struggle to find time for the meetings but also, perhaps paradoxically, limited

For peer review only

For peer review only - http://bmjopen.bmj.com/site/about/guidelines.xhtml

1 time allocated for deliberations during the stakeholder meetings. Finally, sometimes the

For peer review only

For peer review only - http://bmjopen.bmj.com/site/about/guidelines.xhtml

1 difficulty in finding the appropriate representation of stakeholders that the project sought to

For peer review only

For peer review only - http://bmjopen.bmj.com/site/about/guidelines.xhtml

engage was a challenge. In other instances, the problem was the issue of sending a different

For peer review only

1 representative of a group or organization to the meetings each time. Often new people

For peer review only

For peer review only - http://bmjopen.bmj.com/site/about/guidelines.xhtml

struggled to understand the project's background, progress and future aims. Similarly, poor representation of administrative/managerial groups especially from the county which has

> *"R: I can say time…time factor has been… cause most of the time am not usually released from here [hospital x] I try to create my own time, so if you say like am here for the whole day, that means I have to squeeze in 2 shifts, because I usually report here at around 7:30am to 5:30pm so those are 2 shifts, I need to get 2 people to cover my shift but I really don't mind…I really don't mind."*
>
> *R: Yes, you know sometimes we just want to go to another place.*
>
> *M: That is not our office?*
>
> *R: Exactly, if we can be able to see how resources can be able to work for a two day out of the town. So, my issue is I never even participate fully…I am always called to work, so I have to keep rushing. So, I thought at sometimes that if allowable we could actually get out of your offices and we work even though it is one day we actually work until whatever time even if it is midnight. That way I feel it would be more relaxed. I felt that it was a bit tensed and like we need to make this decision, and this is the period we have, and we have to hurry up. I was okay with that speed, but I think at some level maybe we were leaving some other people dragging behind, so could we allocate a bit of time and also out of town."*
> Female Lecturer, Training institution
>
> *"R: The things that were less exciting is that the administration aspect of the project involvement was missing. When I noted that the in charges of the unit or the hospitals were missing in this study, to me I felt your likelihood of sustainability of the good things you have done is questionable and likely to have a challenge. …because there was no commitment from the administration."* Male paediatrician

high staff turnover diminished interest, commitment and ability to follow research activities was perceived by stakeholders as a threat to utility and sustainability

Discussion and Conclusion

Our findings highlight the importance of purposefully selecting stakeholders to fit project needs. Clearly defining roles and expectations for both researchers and the stakeholders, and providing continuous feedback appeared key drivers of meaningful and impactful engagement[38, 39]. Perhaps more vital is mapping the dynamic nature of stakeholder's involvement over a projects' lifetime and creating opportunities to share ideas and views in 'safe' settings. We emphasize the importance of involving across-system actors who are often overlooked in such processes e.g. from frontline health workers who may help articulate and validate the research priorities and as implementors of recommendations to policy makers and regulators with the authority to formalize recommended practices.

We have shown that embedded participation requires investing in social capacity in form of openness of dialogue active listening and courtesy and respectful consideration of ideas contributed. When all elements are present, then participation processes are likely to increase involvement and legitimacy and if participants feel that their views are valued and used, this ultimately enhances how the research may be used in decision making. However, as we learned, participatory processes are complicated by a number of context and structural issues including managing divergent opinions, tensions and mistrust which require interpersonal and facilitation skills which not all academics are trained in or endowed with[40].

Furthermore, there also needs to be more reflection on how to meaningfully measure the worth of embedded participation[41, 42]. This involves including both outcome and process factors and acknowledging that participatory processes typically require long time frames to build awareness and work through existing stakeholder dynamics[43, 44]. There ought to be open discussions on how embedded engagement influences research processes; the significant risks for academics, who are required to adopt practices far from those traditionally taught and having to continuously manage group dynamics. There is need for reviewing funding structures in lieu of conflict between the emergent, dynamic yet invaluable role of engaging stakeholders in research versus strict timelines tied into specified deliverables. Lastly, the need for clearly defined methods for evaluating participation, including focus on power analysis and more studies on developing and applying explanatory theories that better articulate how participation occurs within the relational contexts of coproduction.

We acknowledge: the HSD-N research team, particularly Elizabeth Kyala who helped with archiving the stakeholder engagements and the rest of the HSD-N Collaborative Group who made this work possible. We are also grateful to the health workers, and colleagues

For peer review only - http://bmjopen.bmj.com/site/about/guidelines.xhtml

representing various stakeholder institutions who made this work possible. This paper is submitted with the approval of the Director of KEMRI.

Financial Support - This work was supported by a joint Health Systems Research Initiative grant provided by the Department for International Development, UK (DFID), Economic and Social Research Council (ESRC), Medical Research Council (MRC) and Wellcome Trust, grant number MR/M015386/1. ME is supported by a Wellcome Trust Senior Research Fellowship (#207522). The funding sources had no role in the study design, writing of the report and in the decision to submit the manuscript for publication. This paper is published with the permission of the Director of KEMRI.

Competing interests –JN, CJ, DN and ME received research grants linked to work in Kenya on topics related to this report. The authors have no financial interests to disclose

Contributorship Statement

JN conceived of the idea for the study supported by ME who obtained the funding for this project. Preparation for and conduct of the study was undertaken by JN who also undertook all the interviews, observations and the qualitative analysis with support from ME and DG. CJ provided theoretical support during analysis and write up while ME and DG contributed to the analytical interpretation of the data both in discussion with JN. JN produced the draft manuscript to which all authors contributed to its development. All authors read and approved the final manuscript.

All data relevant to the study are included in this article. Any additional data may not be publicly available due to restrictions. Public availability of data could potentially compromise participant privacy.

References

1. WHO, *Strategy on health policy and systems research: changing the mindset.* 2012.
2. World Health Organization, *Strategy on health policy and systems research: changing the mindset.* 2012.
3. Ghaffar, A., et al., *Strengthening health systems through embedded research.* Bulletin of the World Health Organization, 2017. **95**(2): p. 87.
4. Peters, D.H., et al., *Implementation research: what it is and how to do it.* Bmj, 2013. **347**: p. f6753.
5. Behague, D., et al., *Evidence-based policy-making: the implications of globally-applicable research for context-specific problem-solving in developing countries.* Social Science & Medicine, 2009. **69**(10): p. 1539-1546.
6. English, M., et al., *An intervention to improve paediatric and newborn care in Kenyan district hospitals: understanding the context.* Implementation Science, 2009. **4**(1): p. 42.
7. Cairney, P. and K. Oliver, *Evidence-based policymaking is not like evidence-based medicine, so how far should you go to bridge the divide between evidence and policy?* Health research policy and systems, 2017. **15**(1): p. 1-11.

For peer review only - http://bmjopen.bmj.com/site/about/guidelines.xhtml

For peer review only

8. Langley, J., D. Wolstenholme, and J. Cooke, *'Collective making'as knowledge mobilisation: the contribution of participatory design in the co-creation of knowledge in healthcare.* BMC health services research, 2018. **18**(1): p. 585.
9. Fransman, J., *Charting a course to an emerging field of'research engagement studies': A conceptual meta-synthesis.* Research for All, 2018. **2**(2): p. 185-229.
10. Hanney, S., et al., *Engagement in research: an innovative three-stage review of the benefits for health-care performance.* Health Services and Delivery Research, 2013. **1**(8).
11. Ellen, M.E., et al., *What supports do health system organizations have in place to facilitate evidence-informed decision-making? A qualitative study.* Implementation Science, 2013. **8**(1): p. 84.
12. Boaz, A., et al., *Does the engagement of clinicians and organisations in research improve healthcare performance: a three-stage review.* BMJ open, 2015. **5**(12): p. e009415.
13. Irimu, G., et al., *Developing and introducing evidence based clinical practice guidelines for serious illness in Kenya.* Archives of disease in childhood, 2008. **93**(9): p. 799-804.
14. English, M., *Designing a theory-informed, contextually appropriate intervention strategy to improve delivery of paediatric services in Kenyan hospitals.* Implementation Science, 2013. **8**(1): p. 39.
15. Murphy, G.A., et al., *What capacity exists to provide essential inpatient care to small and sick newborns in a high mortality urban setting?-A cross-sectional study in Nairobi City County, Kenya.* PLoS One, 2018. **13**(4): p. e0196585.
16. Ayieko, P., et al., *A multifaceted intervention to implement guidelines and improve admission paediatric care in Kenyan district hospitals: a cluster randomised trial.* PLoS Med, 2011. **8**(4): p. e1001018.
17. Finlay, L. and B. Gough, *Reflexivity: A practical guide for researchers in health and social sciences*. 2008: John Wiley & Sons.
18. Kohl, E. and P. McCutcheon, *Kitchen table reflexivity: negotiating positionality through everyday talk.* Gender, Place & Culture, 2015. **22**(6): p. 747-763.
19. Irimu, G., et al., *Approaching quality improvement at scale: a learning health system approach in Kenya.* Archives of disease in childhood, 2018. **103**(11): p. 1013-1019.
20. English, M., et al., *Building learning health systems to accelerate research and improve outcomes of clinical care in low-and middle-income countries.* PLoS medicine, 2016. **13**(4): p. e1001991.
21. English, M., et al., *What do we think we are doing? How might a clinical information network be promoting implementation of recommended paediatric care practices in Kenyan hospitals?* Health Research Policy and Systems, 2017. **15**(1): p. 4.
22. Kimberly, J.R. and M.J. Evanisko, *Organizational innovation: The influence of individual, organizational, and contextual factors on hospital adoption of technological and administrative innovations.* Academy of management journal, 1981. **24**(4): p. 689-713.
23. Rebchook, G.M., et al., *Bridging research and practice: Community-researcher partnerships for replicating effective interventions.* AIDS Education and Prevention, 2000. **12**: p. 49-61.
24. Aluvaala, J., et al., *Assessment of neonatal care in clinical training facilities in Kenya.* Archives of disease in childhood, 2015. **100**(1): p. 42-47.
25. Murphy, G.A.V., et al., *Expectations for nursing care in newborn units in Kenya: moving from implicit to explicit standards.* BMJ Global Health, 2018. **3**(2): p. e000645.
26. English, M., et al., *Lessons from a Health Policy and Systems Research programme exploring the quality and coverage of newborn care in Kenya.* BMJ Global Health, 2020. **5**(1).
27. Ministry of Health, *Transforming Health - Accelerating attainment of Health Goals: Kenya Health Sector Strategic Investment Plan (KHSSP) 2013 - 2017*. 2013, Government of Kenya: Nairobi.
28. Nzinga, J., et al., *Exploring the space for task shifting to support nursing on neonatal wards in Kenyan public hospitals.* Human resources for health, 2019. **17**(1): p. 18.

For peer review only - http://bmjopen.bmj.com/site/about/guidelines.xhtml

29. Tsiachristas, A., et al., *Effective coverage and budget implications of skill-mix change to improve neonatal nursing care: an explorative simulation study in Kenya.* BMJ global health, 2019. **4**(6).
30. Oluoch, D., et al., *Neonatal nursing policy and practice in Kenya: Key stakeholders and their views on task-shifting as an intervention to improve care quality.* Wellcome Open Research, 2018. **3**(35): p. 35.
31. Locock, L., et al., *Understanding the role of opinion leaders in improving clinical effectiveness.* Social science & medicine, 2001. **53**(6): p. 745-757.
32. Rycroft-Malone, J., et al., *Implementing health research through academic and clinical partnerships: a realistic evaluation of the Collaborations for Leadership in Applied Health Research and Care (CLAHRC).* Implementation Science, 2011. **6**(1): p. 74.
33. Murphy, G.A.V., et al., *Nairobi Newborn Study: a protocol for an observational study to estimate the gaps in provision and quality of inpatient newborn care in Nairobi City County, Kenya.* BMJ Open, 2016. **6**(12): p. e012448.
34. Gathara, D., et al., *Developing metrics for nursing quality of care for low-and middle-income countries: a scoping review linked to stakeholder engagement.* Human Resources for Health, 2020. **18**: p. 1-16.
35. Fereday, J. and E. Muir-Cochrane, *Demonstrating rigor using thematic analysis: A hybrid approach of inductive and deductive coding and theme development.* International journal of qualitative methods, 2006. **5**(1): p. 80-92.
36. Gioia, D.A., K.G. Corley, and A.L. Hamilton, *Seeking qualitative rigor in inductive research: Notes on the Gioia methodology.* Organizational research methods, 2013. **16**(1): p. 15-31.
37. Murphy, G., et al. *Approach to developing stakeholder-informed and evidence-based task-shifting strategies to improve health services for sick newborns in Kenya*. 2016. Health Systems Global.
38. Guise, J.-M., et al., *A practice-based tool for engaging stakeholders in future research: a synthesis of current practices.* Journal of clinical epidemiology, 2013. **66**(6): p. 666-674.
39. Ray, K.N. and E. Miller, *Strengthening stakeholder-engaged research and research on stakeholder engagement.* Journal of comparative effectiveness research, 2017. **6**(4): p. 375-389.
40. Oliver, K., A. Kothari, and N. Mays, *The dark side of coproduction: do the costs outweigh the benefits for health research?* Health Research Policy and Systems, 2019. **17**(1): p. 33.
41. Lavallee, D.C., et al., *Stakeholder engagement in comparative effectiveness research: how will we measure success?* Journal of Comparative Effectiveness Research, 2012. **1**(5): p. 397-407.
42. Boaz, A., et al., *How to engage stakeholders in research: design principles to support improvement.* Health research policy and systems, 2018. **16**(1): p. 60.
43. Deverka, P.A., et al., *Stakeholder participation in comparative effectiveness research: defining a framework for effective engagement.* Journal of Comparative Effectiveness Research, 2012. **1**(2): p. 181-194.
44. Mathur, V.N., A.D. Price, and S. Austin, *Conceptualizing stakeholder engagement in the context of sustainability and its assessment.* Construction Management and Economics, 2008. **26**(6): p. 601-609.

For peer review only - http://bmjopen.bmj.com/site/about/guidelines.xhtml

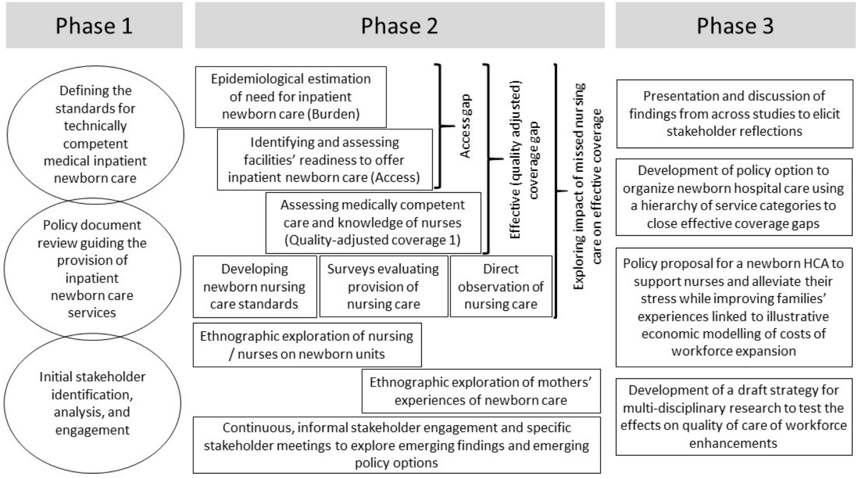

Schematic of HSD-N research components, their inter-relationship and infused stakeholder engagements throughout the research cycle

338x190mm (96 x 96 DPI)

COREQ (COnsolidated criteria for REporting Qualitative research) Checklist

A checklist of items that should be included in reports of qualitative research. You must report the page number in your manuscript where you consider each of the items listed in this checklist. If you have not included this information, either revise your manuscript accordingly before submitting or note N/A.

Topic	Item No.	Guide Questions/Description	Reported on Page No.
**Domain 1: Research team and reflexivity**			
Personal characteristics			
Interviewer/facilitator	1	Which author/s conducted the interview or focus group?	
Credentials	2	What were the researcher's credentials? E.g. PhD, MD	
Occupation	3	What was their occupation at the time of the study?	
Gender	4	Was the researcher male or female?	
Experience and training	5	What experience or training did the researcher have?	
Relationship with participants			
Relationship established	6	Was a relationship established prior to study commencement?	
Participant knowledge of the interviewer	7	What did the participants know about the researcher? e.g. personal goals, reasons for doing the research	
Interviewer characteristics	8	What characteristics were reported about the inter viewer/facilitator? e.g. Bias, assumptions, reasons and interests in the research topic	
**Domain 2: Study design**			
Theoretical framework			
Methodological orientation and Theory	9	What methodological orientation was stated to underpin the study? e.g. grounded theory, discourse analysis, ethnography, phenomenology, content analysis	
Participant selection			
Sampling	10	How were participants selected? e.g. purposive, convenience, consecutive, snowball	
Method of approach	11	How were participants approached? e.g. face-to-face, telephone, mail, email	
Sample size	12	How many participants were in the study?	
Non-participation	13	How many people refused to participate or dropped out? Reasons?	
Setting			
Setting of data collection	14	Where was the data collected? e.g. home, clinic, workplace	
Presence of non-participants	15	Was anyone else present besides the participants and researchers?	
Description of sample	16	What are the important characteristics of the sample? e.g. demographic data, date	
Data collection			
Interview guide	17	Were questions, prompts, guides provided by the authors? Was it pilot tested?	
Repeat interviews	18	Were repeat inter views carried out? If yes, how many?	
Audio/visual recording	19	Did the research use audio or visual recording to collect the data?	
Field notes	20	Were field notes made during and/or after the inter view or focus group?	
Duration	21	What was the duration of the inter views or focus group?	
Data saturation	22	Was data saturation discussed?	
Transcripts returned	23	Were transcripts returned to participants for comment and/or	

For peer review only

Topic	Item No.	Guide Questions/Description	Reported on Page No.
		correction?	
**Domain 3: analysis and findings**			
Data analysis			
Number of data coders	24	How many data coders coded the data?	
Description of the coding tree	25	Did authors provide a description of the coding tree?	
Derivation of themes	26	Were themes identified in advance or derived from the data?	
Software	27	What software, if applicable, was used to manage the data?	
Participant checking	28	Did participants provide feedback on the findings?	
Reporting			
Quotations presented	29	Were participant quotations presented to illustrate the themes/findings? Was each quotation identified? e.g. participant number	
Data and findings consistent	30	Was there consistency between the data presented and the findings?	
Clarity of major themes	31	Were major themes clearly presented in the findings?	
Clarity of minor themes	32	Is there a description of diverse cases or discussion of minor themes?	

Developed from: Tong A, Sainsbury P, Craig J. Consolidated criteria for reporting qualitative research (COREQ): a 32-item checklist for interviews and focus groups. *International Journal for Quality in Health Care*. 2007. Volume 19, Number 6: pp. 349 – 357

Once you have completed this checklist, please save a copy and upload it as part of your submission. DO NOT include this checklist as part of the main manuscript document. It must be uploaded as a separate file.

For peer review only

BMJ Open

The value of Stakeholder Engagement in improving Newborn Care in Kenya: A Qualitative Description of perspectives and lessons learned

Journal:	*BMJ Open*
Manuscript ID	bmjopen-2020-045123.R2
Article Type:	Original research
Date Submitted by the Author:	28-May-2021
Complete List of Authors:	Nzinga, Jacinta; KEMRI-Wellcome Trust Research Programme Nairobi, Health Services Unit<br>Jones, Caroline; KEMRI-Wellcome Trust Research Programme Nairobi, Health Systems Research and Ethics; Oxford University, Nuffield Department of Medicine<br>Gathara, David; KEMRI-Wellcome Trust Research Programme Nairobi, Health Systems Research and Ethics<br>English, Mike; KEMRI-Wellcome Trust Research Programme Nairobi, Health Systems Research and Ethics ; Oxford University, Nuffield Department of Medicine
**Primary Subject Heading**:	Health services research
Secondary Subject Heading:	Health policy, Qualitative research
Keywords:	Organisation of health services < HEALTH SERVICES ADMINISTRATION & MANAGEMENT, Health policy < HEALTH SERVICES ADMINISTRATION & MANAGEMENT, QUALITATIVE RESEARCH, NEONATOLOGY

SCHOLARONE™
Manuscripts

I, the Submitting Author has the right to grant and does grant on behalf of all authors of the Work (as defined in the below author licence), an exclusive licence and/or a non-exclusive licence for contributions from authors who are: i) UK Crown employees; ii) where BMJ has agreed a CC-BY licence shall apply, and/or iii) in accordance with the terms applicable for US Federal Government officers or employees acting as part of their official duties; on a worldwide, perpetual, irrevocable, royalty-free basis to BMJ Publishing Group Ltd ("BMJ") its licensees and where the relevant Journal is co-owned by BMJ to the co-owners of the Journal, to publish the Work in this journal and any other BMJ products and to exploit all rights, as set out in our licence.

The Submitting Author accepts and understands that any supply made under these terms is made by BMJ to the Submitting Author unless you are acting as an employee on behalf of your employer or a postgraduate student of an affiliated institution which is paying any applicable article publishing charge ("APC") for Open Access articles. Where the Submitting Author wishes to make the Work available on an Open Access basis (and intends to pay the relevant APC), the terms of reuse of such Open Access shall be governed by a Creative Commons licence – details of these licences and which Creative Commons licence will apply to this Work are set out in our licence referred to above.

Other than as permitted in any relevant BMJ Author's Self Archiving Policies, I confirm this Work has not been accepted for publication elsewhere, is not being considered for publication elsewhere and does not duplicate material already published. I confirm all authors consent to publication of this Work and authorise the granting of this licence.

For peer review only

The value of Stakeholder Engagement in improving Newborn Care in Kenya: A Qualitative Description of perspectives and lessons learned

Jacinta Nzinga, 1

Caroline Jones, 1, 2

David Gathara, 1

Mike English, 1, 2

Corresponding author: Jacinta Nzinga, KEMRI-Wellcome Trust Research programme, Kenya, PO Box 43640-00100, Nairobi, Kenya. Email: jnzinga@kemri-wellcome.org

1. KEMRI-Wellcome Trust, Nairobi & Kilifi, Kenya
2. Nuffield Department of Medicine, University of Oxford, UK

Key words: stakeholders, participatory research, engagement, health systems,

Word Count

Abstract 291

Main Text 4607

Abstract

Objective: Embedding researchers within health systems results in more socially relevant research and more effective uptake of evidence into policy and practice. However, the practice of embedded health service research remains poorly understood. We explored and assessed the development of embedded participatory approaches to health service research by a health research team in Kenya highlighting the different ways multiple stakeholders were engaged in a neonatal research study.

Methods: We conducted semi-structured qualitative interviews with key stakeholders. Data was analysed thematically using both inductive and deductive approaches.

Setting: Over recent years, the Health Services Unit (HSU) within the KEMRI-Wellcome Trust Research Programme (KWTRP) in Nairobi Kenya, has been working closely with

For peer review only

For peer review only - http://bmjopen.bmj.com/site/about/guidelines.xhtml

organisations and technical stakeholders including, but not limited to, medical and nursing schools, frontline health workers, senior paediatricians, policy makers and county officials, in developing and conducting embedded health research.. This involves researchers embedding themselves in the contexts in which they carry out their research (mainly in county hospitals, local universities and other training institutions), creating and sustaining social networks. Researchers collaboratively worked with stakeholders to identify clinical, operational and behavioural issues related to routine service delivery, formulating and exploring research questions to bring change in practice

Participants: We purposively selected 14 relevant stakeholders spanning policy, training institutions, healthcare workers, regulatory councils and professional associations

Results: The value of embeddedness is highlighted through the description of a recently completed project, Health Services that Deliver for Newborns (HSD-N). We describe how the HSD-N research process contributed to and further strengthened a collaborative research platform and illustrating this project's role in identifying and generating ideas about how to tackle health service delivery problems

Conclusions: We conclude with a discussion about the experiences, challenges and lessons learned regarding engaging stakeholders in the co-production of research

Article Summary

Strengths and Limitation of this study

Strengths from this article include emphasis on involvement; understanding who is and should be involved, when should this engagement occur (i.e., at what points in the research process), and how this engagement should be done (i.e., what are the approaches to engagement that yield the results).

Furthermore, successful participatory processes require; openness of dialogue with a genuine empathy for others' perspectives; active listening and courtesy; early and ongoing voice and creating meaningful decision space throughout the engagement process

However, the limitations of this study include complications by a number of context and resource-based factors including; competing priorities, tension among stakeholder groups, high staff turnover and lack of commitment

There is need for more empiric work to develop and apply explanatory theories, frameworks and models to better understand how participation occurs, under what contextual settings and what is produced

For peer review only - http://bmjopen.bmj.com/site/about/guidelines.xhtml

Introduction

Recent literature has underscored the value of health policy and systems research as an intervention for systems strengthening [1]. In the last decade there has been increased demand for embedded health systems research in low and middle-income countries (LMICs), as leverage for more socially relevant and responsive research, and for more effective uptake of evidence into action/policy/practice[2, 3]. Further, implementation research has highlighted the need for context-specific research evidence as part of solutions to address the translation of knowledge into practice[4-6].However, the uptake of research findings heavily depends on the credibility of the information produced which is in turn dependent on trusted local stakeholders' expertise and their active, meaningful involvement throughout the research process [7-9].

This paper provides a brief description of our (a health research group) history of more than 15 years of engaging with stakeholders and conducting health services research in Kenyan hospitals and explores the relational and organisational processes underlying network activities; examining the spaces in which stakeholder engagement occurred over a number of years during work which focused on hospital improvement [10-12]. It then provides a critical analysis of the most recent lessons learnt through a description of a study aimed at understanding how local structural, contextual and cultural factors influenced the research-policy-practice engagement process in a recently completed health systems research project. The aim is to provide a better understanding of the requirements of embedded participation in responding to local problems.

Study background

The Health Services Unit (HSU) of the KEMRI-Wellcome Trust Research Programme started working closely with the Ministry of Health (MoH) of Kenya in 2004 developing and implementing research on facility-based care to improve child and newborn survival [13-15]. Early work focused on developing and implementing a multifaceted intervention aimed at improving paediatric inpatient care in district hospitals in Kenya [16]. Data collection included long-term participant observation and continuous reflection on the positionality of study team members embedded in the study hospitals [17, 18]. To allow engagement with stakeholders, regular evidence synthesis meetings and feedback meetings were held with the hospitals. There were bi-monthly phone calls to understand how the intervention was unfolding as well as formal and informal discussions and consultations with the stakeholders to understand their interest in the engagement. A key lesson from the project was that

For peer review only - http://bmjopen.bmj.com/site/about/guidelines.xhtml

changing practice and system hospitals required specific collaboration with partners who are usually considered the subjects of research.

Consequently, driven by the need for system wide improvement, the HSU partnered with the MoH, the Kenyan Paediatric Association and 14 county (district) level hospitals in 2010 to create a Clinical Information Network (CIN) spread over 16 counties in eastern, western and central Kenya [19]. The network aimed to produce high-quality process and outcome data from individual admissions to paediatric wards in Kenyan hospitals and use these data to inform improvement strategies. Through collaborative working, the network has grown into a community of practice aimed at slowly changing hospital culture through sustained engagement, peer support and linking hospitals within the network [20]. The effects of the CIN platform, critically explored through formative explanation and theory of change, are documented elsewhere [21].

Through these projects, the research team began to learn from stakeholders how contexts shape service delivery, and how relationships between the research team, health managers and health workers develop and shape the delivery of the interventions over time[22, 23].However, this research process involved limited true co-production, partly because research funding provided limited support for extensive work of this kind. Furthermore, it was apparent that the practice of embedded Health Policy and Systems Research (HPSR) in LMICs was, at that time, not very well defined and that trial-and-error strategies like our own were often applied.

Over time, the research group developed a more deliberate and collaborative approach that was taken forward in subsequent projects including the HSD-N project detailed below.

The HSD-N project: 2013-2018

As a research team, concerned by the high neonatal mortality in Nairobi, we held consultative meetings with the County Government of Nairobi and other key stakeholders. Together, and whilst drawing on our 10 years' research experience on quality of care[24-26] we co-developed the HSD-N project with key stakeholders. The project aimed to address the challenges influencing the delivery of essential inpatient newborn services in Nairobi County with a particular focus on nursing care, which was highlighted by all stakeholders as a neglected topic (*figure 1*).

The initial approach to conceptualising how gaps might be addressed was informed by Kenyan policy objectives, specifically the focus at national policy level on task shifting [27] and early discussions with the Nairobi City Council (NCC) in which concerns over how

For peer review only - http://bmjopen.bmj.com/site/about/guidelines.xhtml

newborn care was delivered across the public, private and faith-based sectors were raised. In light of the prevailing policy environment our research included an explicit aim to explore the potential of task shifting through the use of health care assistants (HCAs) to support nursing care as one potentially important component for improved newborn care practice in Kenyan and possibly other LMICs [28, 29].

The HSD-N project took place in three phases (*figure 1*). At the heart of this work, was a strategic approach to researching and intervening in the health system based on collaborative engagement from the outset. Building on relationships developed from previous projects we began to forge new linkages with powerful (had authority to influence key policy decisions in newborn care) professionals including regulators, health professional bodies, private institutions and other major decisions makers in health in Kenya[30]. This stakeholder network was a core facilitator for truly collaborative and co-produced research.

Phase 1 (2014-2015): The existing links developed by the HSU over the years allowed an initial drafting of a list of key stakeholders likely to play a critical role in the conduct and impact of research addressing nursing service policy and practice issues [31, 32]. The list was collaboratively reviewed by the research team and initial stakeholders with more stakeholders added following certain strategic considerations. These included: the projects' core research questions; the power and interests of those who would be responsible for making decisions informed by the research; and the individuals and groups that would be affected by such decisions. Specifically, during stakeholder meetings, the appropriateness and effectiveness of the research approach adopted was heavily dependent on learning from and listening to these stakeholders.

Phase 2 (2015-2017): The empirical data collection for the HSD-N project started with two distinct bodies of work see Fig 1[15, 33].). During this empirical phase of the project, engagement activities included stakeholder engagement meetings and workshops, various trainings and hospital feedback meetings on empirical findings (Table 1).

Phase 3 (2017-2018): Alongside empirical data collection a series of stakeholder workshops with nursing and neonatal care experts helped define core standards for care of sick newborns in Kenyan hospitals [25, 34]. The stakeholder workshops focused on: the capacity required to provide an essential package of services for sick newborns; understanding the nursing time/skills needed for effective delivery of interventions and were complimented by hospital feedback meetings and various topic-specific meetings as shown in Table 1.

For peer review only - http://bmjopen.bmj.com/site/about/guidelines.xhtml

(Figure 1 about here) Fig 1: Schematic of HSD-N research components, their inter-relationship and infused stakeholder engagements throughout the research cycle

To provide an in-depth understanding of how the HSD-N project was developed and implemented in practice, we present a chronological timeline of the research process and how the 'engagement platform' developed, identifying the key engagement activities that were influential in enabling coproduction during the lifetime of the project (*Table 1*).

Table 1*: *chronological representation of research engagement and contribution of the HSD-N project in shaping engagement and co-production of research

YEAR	2014	2015	2016	2017	2018
**ENGAGEMENT PLATFORMS**					
**Meetings**	2 meetings with representatives from the Nairobi County health management team, with the universities, KP and MoH. These meetings were held during the drafting of the proposal through to submission for funding	1 Meeting with County Executive Member for Health Services<br><br>Stakeholder meetings introduction to the HSD-N project<br><br>Meeting on estimating the Requirement for Inpatient Neonatal Care and Neonatal Burden of Disease	Expert meeting on developing Neonatal Nursing Standards of Practice<br><br>Stakeholder meetings on<br><br>Estimating the Requirement for Inpatient Neonatal Care<br><br>Basic standards of quality newborn care<br><br>Results of the Nairobi newborn study on neonatal service provision	Nairobi Newborn Study feedback and presentation of report meeting<br><br>Feedback meeting on results on the context issues for neonatal nursing task shifting<br><br>Hospital specific feedback meetings on task sharing in practice<br><br>An introduction to survey work on missed	Healthcare assistants costing meeting<br><br>Cross-site Hospital feedback meetings on task sharing in practice<br><br>Developing nursing indicators meeting<br><br>Feedback on missed care survey work meeting

For peer review only - http://bmjopen.bmj.com/site/about/guidelines.xhtml

				neonatal care meeting	
**Workshops**		Checking newborn epidemiological estimates with newborn experts<br><br>Check the facilities we identified for the survey<br><br>Disseminate the facility survey findings	'Fact-check' workshop on the early facility survey findings<br><br>Expert workshop meeting on developing Neonatal Nursing Standards of Practice	Two workshops on NHCA scope of practice and training,<br><br>On hierarchical task analysis (two of these)<br><br>On nursing missed care questionnaire design	One on levels of neonatal care<br><br>One on costing.
**Interviews**		Stakeholder mapping and collecting views on task-shifting with pediatric and nursing experts, academic stakeholders			End of project interviews with 14 stakeholders
**Training**			Hierarchical Task Analysis meeting	Missed care observational methods training	
**Hospital specific feedback meetings**		All through			
**Multi-disciplinary quarterly researcher reflective meetings**		All through			

For peer review only

Methods

Research Design

This was a qualitative exploratory study

Study setting

To explore the content and consequences of the HSD-N engagement activities over the project period, we conducted key informant interviews and pre-planned observation of HSD-N meetings within Nairobi County.

Ethics Approval

Ethical approval was obtained from the Kenya Medical Research Institute Ethical Review Committee (Approval number SERU 3366). Written informed consent was obtained from all the participants

Patient and public involvement

Patients were not involved in setting the research question or the outcome measures, but key public stakeholders who were part of the HSD-N collaborative group and described in this paper were consulted in the design, conduct and dissemination of the study findings.

Key informant interviews

To build our understanding of how neonatal care is perceived within policy and practice environments we tracked the continuing purposeful engagement with stakeholders exploring the influence of stakeholder-researcher interactions. Six months before the end of the project we conducted in-depth interviews with purposively selected key informants with potential policy influence, including: The Nursing Council of Kenya (NCK), National Nursing Association of Kenya (NNAK), Kenya Pediatric Association (KPA), various nursing training schools, private organizations, and frontline workers. Selected participants included both males and females, with varied years of working experience and with specific expertise in newborn care. Although the HSD-N project was geographically Nairobi focused, many of the groups represented national level stakeholders.

The interviews were guided by a pilot tested interview guide that focused on what drove individuals to be part of the stakeholder network, their understanding of the project, nature of involvement, how their inputs were gathered and any impact of their involvement. All interviews were conducted in English, within participants' work premises and lasted 40mins- 60mins. The interviews were audio-recorded following informed consent from participants and field notes taken during and after the interviews.

For peer review only - http://bmjopen.bmj.com/site/about/guidelines.xhtml

Data analysis

Data were analysed both inductively (emerging from the interview data and observation notes) and deductively driven by a priori themes and coded using Nvivo Qualitative software. Data was coded around the purpose and mechanisms of engagement, researcher-stakeholder relationships, and how local structural, contextual and cultural factors influenced the process of research-policy-practice engagement [35, 36].Through critical analysis of the empirical data and reflexivity we developed a rich description of the concerns and interests of stakeholders likely to be affected by the research findings. The findings are summarised under four main themes: classification and description of stakeholders; interpreting the HSD-N engagement; barriers and facilitators of engagement and the context and nature of engagement.

Results

The results we present are based on interviews with 14 selected stakeholders at the end of the HSD-N project in 2018 and presented under 4 main themes (*see Table 2*)

Table 2. Description of the emerging themes and sub-themes

	THEMES	SUB-THEMES
1.	Classification and description of stakeholders	Stakeholder identification process
		Nature of engagement
		Level of engagement
2.	Context and nature of engagement	Perceived value of stakeholder meetings
		Role of feedback in shaping engagement
		Strategies used in managing voices of the various stakeholders
3.	Interpreting the HSD-N engagement	Technical capacity to engage with various research topics
		Ability to implement lessons from research project

For peer review only - http://bmjopen.bmj.com/site/about/guidelines.xhtml

4.	Facilitator and barriers of the engagement	Early engagement in the project
		Creating safe spaces for deliberations
		Multi-level actor engagement
		Stakeholders' competing priorities
		Perceived 'poor' compensation
		High stakeholder turn-over

Classification and description of stakeholders

Stakeholders of the HSD-N project were primarily from the public sector which provides the majority of neonatal care in Nairobi [37]. However, some stakeholders from private and non-for-profit organizations were included. None of the stakeholders were compensated for their time on the project although there were in-built mechanisms to build capacity through short trainings on research and select relevant quality improvement topics. The roles of stakeholders in the HSD-N project was linked to 4 key project activities (*table 3):* i) study planning (includes co-design of the research questions; ii) study design procedures and development of study tools); iii) study implementation (as study participants, development of modelling scenarios or training curricula, and drafting nursing standards) and iv) interpretation and translation (ambassadors of implementation and change).

> *"R: This one [HSD-N] was different thing …. in the initial phases of the design of the project we were involved as part of the team that we were actually designing the tools and refining them and even having consensus. So, this was good… because I participated more." Female senior university lecturer*
>
> *"I collected some data, they involved me in data collection on task sharing and I felt well… I felt engaged, like I can actually give people who are here, who work in Kenyatta and get their views" Female nurse manager*

For peer review only - http://bmjopen.bmj.com/site/about/guidelines.xhtml

To fully understand who should be engaged, when should this engagement occur (i.e., at what points in the research process), we explored the nature of the various engagements and present in Table 3

For peer review only

For peer review only - http://bmjopen.bmj.com/site/about/guidelines.xhtml

Table 3: Description and roles of HSDN stakeholders

STAKEHOLDER CATEGORIES	Policy maker	Regulator	Professional association	Training institutions	Health managers	Health workers	Researchers
	Department of monitoring and evaluation<br>Department of Nursing services, Ministry of Health (MOH)<br>World Health Organization (WHO)<br>United Nations International Children's Fund (UNICEF)	Nursing council of Kenya (NCK)	Kenya Paediatric Association (KPA)<br><br>The National Nursing Association of Kenya (NNAK)	Kenya Medical Training College (KMTC)<br><br>University of Nairobi (UON)<br><br>AgaKhan University Hospital (AKUH)<br><br>Kenyatta University (KU)	Ward and departmental managers of;<br><br>Public hospitals<br>Mission hospitals<br>Private hospitals	Nurses, medical officers and clinical officers of;<br><br>Public hospitals<br>Mission hospitals<br>Private hospitals	Multi-disciplinary team of researchers from;<br><br>(Kenya Medical Research Institute-Wellcome Trust Research Programme (KEMRI-WTRP), AgaKhan University Hospital (AKUH), Strathmore University<br>Oxford University<br>Warwick University
**NATURE OF ENGAGEMENT**							
**Consultative**	Collaborated with the team in study design, implementation<br>Advised on the political and regulatory landscape	Collaborated with the team by offering advice on study implementation.<br>Advised on the political and	Advised on the political and regulatory landscape	Provided technical theoretical and practical advice during various sessions of evidence generation	Provided technical advice during various sessions of evidence generation<br><br>Significant voice in shaping NHCA roles (some were already using helpers informally or in	Provided technical advice during various sessions of evidence generation and reflective of the practical realities in routine service provision	

For peer review only - http://bmjopen.bmj.com/site/about/guidelines.xhtml

		regulatory landscape		Major voice in design of neonatal health care assistants (NHCA) scope of work and preliminary curriculum plus potential salary	private sector more formally) and also suggestions on the political presentation of the NHCA cadre<br><br>Useful reflections on the practical realities in routine service provision			
**Involved**			Involved in aspects of study implementation, including data collection<br><br>Offered expert critique and suggestions on improving emerging findings (e.g. neonatal burden estimation)				Involved in aspects of study implementation, including data collection	Mainly involved in evidence generation, incorporating the technical advice of various stakeholders in the analysis<br><br>Collating the interpretation of findings and implications on policy and practice
**Interpretation and translation**								
**Strategic endorsement**	Added credibility to the research evidence and enabled other big	Added credibility to the research evidence and	Acted as ambassadors of change and					

For peer review only - http://bmjopen.bmj.com/site/about/guidelines.xhtml

	players to be part of the deliberations (e.g. NNAK, NCK)<br><br>Statutory agreement of translating study findings into policy recommendations	enabled other big players to be part of the deliberations (e.g. NNAK, NCK)<br><br>Offered reflections on feasibility of translating evidence into practice	implementation of study findings				

For peer review only

For peer review only - http://bmjopen.bmj.com/site/about/guidelines.xhtml

Context and nature of engagement processes

In table 3 above, we provided a categorization of stakeholders, the nature of engagement and stakeholders' perceived roles in the project over the 4-year implementation period.

We also sought stakeholder's opinions as to why they think they were invited to be part of this project and why they continued engaging with the project activities. Most participants reported they believed they had important contributions to make and that the project allowed an avenue for this while others joined out of personal interest:

> *"R: Personally, I love something that is out of what I do every day... like research can help in boosting, ... I can change in the unit...I love doing different things from the norm that is why I felt I can be part of this. This project is beyond relevant... because our unit is.... we handle 200 babies and it is like 50% will go 50% will die. You know if are in such a project ...you can do something about the situation... well I believe it is very relevant."* Male paediatrician
>
> *"R: Well, there is always the person part of it [HSDN] that you interact with people because quite often when we are working, everybody is just too busy to interact with each other"* Female paediatrician

As mentioned above, the HSDN project ran several activities as part of stakeholder engagement using concept mapping and focus groups, and all these activities were documented and archived to inform the process and success of the project. (*refer to Table 1 for type and purpose of meeting*). Stakeholders described these meetings as useful 'engagement spaces' that provided opportunity to not only discuss various aspects of the research but to also get updates regarding the project and included learning opportunities.

Particularly valued was provision of regular feedback, ensuring that the most knowledgeable stakeholders in the subject matter were present and that their views were sought and incorporated into the final reports. Feedback meetings allowed researchers to check understanding and modify interpretations and key messages. In particular, efforts by the research team to understand why there may be support or resistance to some of the potential recommendations was also important.

For peer review only - http://bmjopen.bmj.com/site/about/guidelines.xhtml

However, during these meetings it was not always easy managing differing views and reactions regarding emerging recommendations, and it was particularly challenging dealing with the varied power dynamics from different groups and individuals. However, we observed stakeholders' free and frank exchanges in voicing opinions, open disagreement and on occasions the research team taking on arbitration roles to ensure all voices were heard. During interviews, stakeholders recounted the various strategies they drew on in making sure they were heard and in respectfully disagreeing with opinions as illustrated below, The nature of engagement that emerged was mainly both consultative and collaborative which enabled the cumulation of understanding and development of meaningful relationships.

Interpreting the HSD-N engagement

We were interested in the stakeholders' articulation of how research findings were established and their influence over such findings as this would potentially benefit effective implementation.

"R: In the meetings there are those people who participated in the research projects and also in the meetings, so it gave the project authority. and it made sense to the people who participated. When we hear that those who participated are also here, we also appreciate that report and the feedback and the evidence that is being presented." Male, Professional association

"I think was a very exciting journey because we were able to share with each other, with the paediatric association, to discuss with the paediatricians and even have the consensus of where we need to be. I also I think the other exciting journey came in when I was involved as part of the cohort to do the publication." Female, Regulatory Council

"R: If they are not listening then you still continue shouting there is no other language but of course occasionally you have to sit down think of another strategy. In such a situation that is the time when you think of who else has a voice, you have to think of who else could be having the same mind as mine so that you put the two voices together and we see whether we can be heard that is one strategy." Female frontline nurse

For peer review only - http://bmjopen.bmj.com/site/about/guidelines.xhtml

During the interviews, we reflected with stakeholders about; i) their technical capacity and ability to engage with the varied research topics ii) how their feedback was incorporated into the project and iii) ability to implement lessons from the project. Examples are provided below;

On ability to conceptually engage with the research, with experiential understanding of the research problem, stakeholder reported the importance of having technical capacity to engage and also felt that their feedback influenced the research process Furthermore, stakeholders who had the ability, described application of new clinical information in their hospitals

> *"I also participated in the review of the procedure manual so I knew the procedures and when you tell me that a nurse assistant will be able to give fluids or to do blood transfusion then am going back to the rationale of that procedure"* Female nurse manager
>
> *"Just the voice, convincing people that it is worth taking it up, and the fact that I am a trainer… I understand all curriculum and I understand the needs in the service delivery units I think with that in mind it [engagement] has enabled me to work with whoever towards achieving the goals of the project.*" Female lecturer, training college
>
> *"R: Every time we came out of the meetings we would also come and improve things within the facility. So, there is already been a positive feedback and in fact use of the learning that we have done within the facilities."* Female Paediatrician

According to the stakeholders, the process of cultivating long term researcher-stakeholder relationships meant respecting each other's time and commitment, continuously reviving interest in the project and clearly communicating and negotiating expectations.

Barriers and facilitators of the HSD-N engagement process

We learned to be sensitive to stakeholders' time commitments as this was perceived as highly important for continued engagement. Understanding how stakeholder integrate on-going research activities into daily work enabled bringing together people from various levels of the

For peer review only - http://bmjopen.bmj.com/site/about/guidelines.xhtml

health sector building multi-layered perspectives of the research project in terms of its implementation

As a research team, we learned that successful stakeholder engagement required early involvement in project design, providing pre-readings to enable informed discussion, creatively using "icebreakers," especially when engaging stakeholders with differing experiences/perspectives and clearly communicating the anticipated commitment of time and level of engagement.

> *"R: That [stakeholder engagement] kind of interaction has been quite good. Quite often when the team sent out mail, some of us try to say okay 'I have been sent this and I think I need to meet my obligation'. That communication I think it has been quite good. And top of that, it hasn't been overwhelming because for this project we have been given adequate time to be able to address things and of course most of those documents they have been sending have not been these huge heavy documents that bog one down"* Female lecturer, training institution

> *"R: The meetings were fairly regular and fairly spaced …so would have like once in six months, so I think the regularity was good because most people are really pressed on time"* Female, frontline nurse
>
> *"R: I realized we are meeting with a variety of stakeholders, from different facilities, that is terms of the levels public, private and then we have lecturers, we have doctors and the Nursing Council. I think it's a good way because they are able to listen to us the people on the lower level. What we are going through…, they were able really to compare and see actually this is something that will work."* Female, Professional Association
>
> *"R: The study reports are available for most of us… we are able to go through the whole process of the study we are able to go through and it is available, so I think that is also a strong area for the study group."* Male, training institution

For peer review only - http://bmjopen.bmj.com/site/about/guidelines.xhtml

For peer review only

For peer review only - http://bmjopen.bmj.com/site/about/guidelines.xhtml

1 Despite the positive feedback, the engagement over time also had some limitations. The most

For peer review only

For peer review only - http://bmjopen.bmj.com/site/about/guidelines.xhtml

commonly reported barriers included competing priorities by most of the stakeholders and

For peer review only

For peer review only - http://bmjopen.bmj.com/site/about/guidelines.xhtml

1 therefore a struggle to find time for the meetings but also, perhaps paradoxically, limited

For peer review only

For peer review only - http://bmjopen.bmj.com/site/about/guidelines.xhtml

time allocated for deliberations during the stakeholder meetings. Finally, sometimes the

For peer review only

For peer review only - http://bmjopen.bmj.com/site/about/guidelines.xhtml

1 difficulty in finding the appropriate representation of stakeholders that the project sought to

For peer review only

For peer review only - http://bmjopen.bmj.com/site/about/guidelines.xhtml

engage was a challenge. In other instances, the problem was the issue of sending a different

For peer review only - http://bmjopen.bmj.com/site/about/guidelines.xhtml

representative of a group or organization to the meetings each time. Often new people

For peer review only

For peer review only - http://bmjopen.bmj.com/site/about/guidelines.xhtml

struggled to understand the project's background, progress and future aims. Similarly, poor representation of administrative/managerial groups especially from the county which has

> *"R: I can say time…time factor has been… cause most of the time am not usually released from here [hospital x] I try to create my own time, so if you say like am here for the whole day, that means I have to squeeze in 2 shifts, because I usually report here at around 7:30am to 5:30pm so those are 2 shifts, I need to get 2 people to cover my shift but I really don't mind…I really don't mind."*
>
> *R: Yes, you know sometimes we just want to go to another place.*
>
> *M: That is not our office?*
>
> *R: Exactly, if we can be able to see how resources can be able to work for a two day out of the town. So, my issue is I never even participate fully…I am always called to work, so I have to keep rushing. So, I thought at sometimes that if allowable we could actually get out of your offices and we work even though it is one day we actually work until whatever time even if it is midnight. That way I feel it would be more relaxed. I felt that it was a bit tensed and like we need to make this decision, and this is the period we have, and we have to hurry up. I was okay with that speed, but I think at some level maybe we were leaving some other people dragging behind, so could we allocate a bit of time and also out of town."*
> Female Lecturer, Training institution
>
> *"R: The things that were less exciting is that the administration aspect of the project involvement was missing. When I noted that the in charges of the unit or the hospitals were missing in this study, to me I felt your likelihood of sustainability of the good things you have done is questionable and likely to have a challenge. …because there was no commitment from the administration."* Male paediatrician

high staff turnover diminished interest, commitment and ability to follow research activities was perceived by stakeholders as a threat to utility and sustainability

For peer review only - http://bmjopen.bmj.com/site/about/guidelines.xhtml

Discussion and Conclusion

Our findings highlight the importance of purposefully selecting stakeholders to fit project needs. Clearly defining roles and expectations for both researchers and the stakeholders, and providing continuous feedback appeared key drivers of meaningful and impactful engagement[38, 39]. Perhaps more vital is mapping the dynamic nature of stakeholder's involvement over a projects' lifetime and creating opportunities to share ideas and views in 'safe' settings. We emphasize the importance of involving across-system actors who are often overlooked in such processes e.g. from frontline health workers who may help articulate and validate the research priorities and as implementors of recommendations to policy makers and regulators with the authority to formalize recommended practices.

We have shown that embedded participation requires investing in social capacity in form of openness of dialogue active listening and courtesy and respectful consideration of ideas contributed. When all elements are present, then participation processes are likely to increase involvement and legitimacy and if participants feel that their views are valued and used, this ultimately enhances how the research may be used in decision making. However, as we learned, participatory processes are complicated by a number of context and structural issues including managing divergent opinions, tensions and mistrust which require interpersonal and facilitation skills which not all academics are trained in or endowed with[40].

Furthermore, there also needs to be more reflection on how to meaningfully measure the worth of embedded participation[41, 42]. This involves including both outcome and process factors and acknowledging that participatory processes typically require long time frames to build awareness and work through existing stakeholder dynamics[43, 44]. There ought to be open discussions on how embedded engagement influences research processes; the significant risks for academics, who are required to adopt practices far from those traditionally taught and having to continuously manage group dynamics. There is need for reviewing funding structures in lieu of conflict between the emergent, dynamic yet invaluable role of engaging stakeholders in research versus strict timelines tied into specified deliverables. Lastly, the need for clearly defined methods for evaluating participation, including focus on power analysis and more studies on developing and applying explanatory theories that better articulate how participation occurs within the relational contexts of coproduction.

We acknowledge: the HSD-N research team, particularly Elizabeth Kyala who helped with archiving the stakeholder engagements and the rest of the HSD-N Collaborative Group who made this work possible. We are also grateful to the health workers, and colleagues

For peer review only - http://bmjopen.bmj.com/site/about/guidelines.xhtml

representing various stakeholder institutions who made this work possible. This paper is submitted with the approval of the Director of KEMRI.

Financial Support - This work was supported by a joint Health Systems Research Initiative grant provided by the Department for International Development, UK (DFID), Economic and Social Research Council (ESRC), Medical Research Council (MRC) and Wellcome Trust, grant number MR/M015386/1. ME is supported by a Wellcome Trust Senior Research Fellowship (#207522). The funding sources had no role in the study design, writing of the report and in the decision to submit the manuscript for publication. This paper is published with the permission of the Director of KEMRI.

Competing interests –JN, CJ, DN and ME received research grants linked to work in Kenya on topics related to this report. The authors have no financial interests to disclose

Contributorship Statement

JN conceived of the idea for the study supported by ME who obtained the funding for this project. Preparation for and conduct of the study was undertaken by JN who also undertook all the interviews, observations and the qualitative analysis with support from ME and DG. CJ provided theoretical support during analysis and write up while ME and DG contributed to the analytical interpretation of the data both in discussion with JN. JN produced the draft manuscript to which all authors contributed to its development. All authors read and approved the final manuscript.

All data relevant to the study are included in this article. Any additional data may not be publicly available due to restrictions. Public availability of data could potentially compromise participant privacy.

Data Availability Statement

No additional data available

References

1. WHO, *Strategy on health policy and systems research: changing the mindset*. 2012.
2. World Health Organization, *Strategy on health policy and systems research: changing the mindset*. 2012.
3. Ghaffar, A., et al., *Strengthening health systems through embedded research.* Bulletin of the World Health Organization, 2017. **95**(2): p. 87.
4. Peters, D.H., et al., *Implementation research: what it is and how to do it*. Bmj, 2013. **347**: p. f6753.

For peer review only

5. Behague, D., et al., *Evidence-based policy-making: the implications of globally-applicable research for context-specific problem-solving in developing countries.* Social Science & Medicine, 2009. **69**(10): p. 1539-1546.

6. English, M., et al., *An intervention to improve paediatric and newborn care in Kenyan district hospitals: understanding the context*. Implementation Science, 2009. **4**(1): p. 42.

7. Cairney, P. and K. Oliver, *Evidence-based policymaking is not like evidence-based medicine, so how far should you go to bridge the divide between evidence and policy?* Health research policy and systems, 2017. **15**(1): p. 1-11.

8. Langley, J., D. Wolstenholme, and J. Cooke, *'Collective making'as knowledge mobilisation: the contribution of participatory design in the co-creation of knowledge in healthcare*. BMC health services research, 2018. **18**(1): p. 585.

9. Fransman, J., *Charting a course to an emerging field of'research engagement studies': A conceptual meta-synthesis*. Research for All, 2018. **2**(2): p. 185-229.

10. Hanney, S., et al., *Engagement in research: an innovative three-stage review of the benefits for health-care performance*. Health Services and Delivery Research, 2013. **1**(8).

11. Ellen, M.E., et al., *What supports do health system organizations have in place to facilitate evidence-informed decision-making? A qualitative study*. Implementation Science, 2013. **8**(1): p. 84.

12. Boaz, A., et al., *Does the engagement of clinicians and organisations in research improve healthcare performance: a three-stage review*. BMJ open, 2015. **5**(12): p. e009415.

13. Irimu, G., et al., *Developing and introducing evidence based clinical practice guidelines for serious illness in Kenya*. Archives of disease in childhood, 2008. **93**(9): p. 799-804.

14. English, M., *Designing a theory-informed, contextually appropriate intervention strategy to improve delivery of paediatric services in Kenyan hospitals.* Implementation Science, 2013. **8**(1): p. 39.

15. Murphy, G.A., et al., *What capacity exists to provide essential inpatient care to small and sick newborns in a high mortality urban setting?-A cross-sectional study in Nairobi City County, Kenya*. PLoS One, 2018. **13**(4): p. e0196585.

16. Ayieko, P., et al., *A multifaceted intervention to implement guidelines and improve admission paediatric care in Kenyan district hospitals: a cluster randomised trial.* PLoS Med, 2011. **8**(4): p. e1001018.

17. Finlay, L. and B. Gough, *Reflexivity: A practical guide for researchers in health and social sciences*. 2008: John Wiley & Sons.

For peer review only - http://bmjopen.bmj.com/site/about/guidelines.xhtml

18. Kohl, E. and P. McCutcheon, *Kitchen table reflexivity: negotiating positionality through everyday talk*. Gender, Place & Culture, 2015. **22**(6): p. 747-763.

19. Irimu, G., et al., *Approaching quality improvement at scale: a learning health system approach in Kenya*. Archives of disease in childhood, 2018. **103**(11): p. 1013-1019.

20. English, M., et al., *Building learning health systems to accelerate research and improve outcomes of clinical care in low-and middle-income countries*. PLoS medicine, 2016. **13**(4): p. e1001991.

21. English, M., et al., *What do we think we are doing? How might a clinical information network be promoting implementation of recommended paediatric care practices in Kenyan hospitals?* Health Research Policy and Systems, 2017. **15**(1): p. 4.

22. Kimberly, J.R. and M.J. Evanisko, *Organizational innovation: The influence of individual, organizational, and contextual factors on hospital adoption of technological and administrative innovations*. Academy of management journal, 1981. **24**(4): p. 689-713.

23. Rebchook, G.M., et al., *Bridging research and practice: Community-researcher partnerships for replicating effective interventions*. AIDS Education and Prevention, 2000. **12**: p. 49-61.

24. Aluvaala, J., et al., *Assessment of neonatal care in clinical training facilities in Kenya*. Archives of disease in childhood, 2015. **100**(1): p. 42-47.

25. Murphy, G.A.V., et al., *Expectations for nursing care in newborn units in Kenya: moving from implicit to explicit standards*. BMJ Global Health, 2018. **3**(2): p. e000645.

26. English, M., et al., *Lessons from a Health Policy and Systems Research programme exploring the quality and coverage of newborn care in Kenya*. BMJ Global Health, 2020. **5**(1).

27. Ministry of Health, *Transforming Health - Accelerating attainment of Health Goals: Kenya Health Sector Strategic Investment Plan (KHSSP) 2013 - 2017*. 2013, Government of Kenya: Nairobi.

28. Nzinga, J., et al., *Exploring the space for task shifting to support nursing on neonatal wards in Kenyan public hospitals*. Human resources for health, 2019. **17**(1): p. 18.

29. Tsiachristas, A., et al., *Effective coverage and budget implications of skill-mix change to improve neonatal nursing care: an explorative simulation study in Kenya*. BMJ global health, 2019. **4**(6).

For peer review only - http://bmjopen.bmj.com/site/about/guidelines.xhtml

30. Oluoch, D., et al., *Neonatal nursing policy and practice in Kenya: Key stakeholders and their views on task-shifting as an intervention to improve care quality.* Wellcome Open Research, 2018. **3**(35): p. 35.

31. Locock, L., et al., *Understanding the role of opinion leaders in improving clinical effectiveness.* Social science & medicine, 2001. **53**(6): p. 745-757.

32. Rycroft-Malone, J., et al., *Implementing health research through academic and clinical partnerships: a realistic evaluation of the Collaborations for Leadership in Applied Health Research and Care (CLAHRC).* Implementation Science, 2011. **6**(1): p. 74.

33. Murphy, G.A.V., et al., *Nairobi Newborn Study: a protocol for an observational study to estimate the gaps in provision and quality of inpatient newborn care in Nairobi City County, Kenya.* BMJ Open, 2016. **6**(12): p. e012448.

34. Gathara, D., et al., *Developing metrics for nursing quality of care for low-and middle-income countries: a scoping review linked to stakeholder engagement.* Human Resources for Health, 2020. **18**: p. 1-16.

35. Fereday, J. and E. Muir-Cochrane, *Demonstrating rigor using thematic analysis: A hybrid approach of inductive and deductive coding and theme development.* International journal of qualitative methods, 2006. **5**(1): p. 80-92.

36. Gioia, D.A., K.G. Corley, and A.L. Hamilton, *Seeking qualitative rigor in inductive research: Notes on the Gioia methodology.* Organizational research methods, 2013. **16**(1): p. 15-31.

37. Murphy, G., et al. *Approach to developing stakeholder-informed and evidence-based task-shifting strategies to improve health services for sick newborns in Kenya.* 2016. Health Systems Global.

38. Guise, J.-M., et al., *A practice-based tool for engaging stakeholders in future research: a synthesis of current practices.* Journal of clinical epidemiology, 2013. **66**(6): p. 666-674.

39. Ray, K.N. and E. Miller, *Strengthening stakeholder-engaged research and research on stakeholder engagement.* Journal of comparative effectiveness research, 2017. **6**(4): p. 375-389.

40. Oliver, K., A. Kothari, and N. Mays, *The dark side of coproduction: do the costs outweigh the benefits for health research?* Health Research Policy and Systems, 2019. **17**(1): p. 33.

41. Lavallee, D.C., et al., *Stakeholder engagement in comparative effectiveness research: how will we measure success?* Journal of Comparative Effectiveness Research, 2012. **1**(5): p. 397-407.

For peer review only

For peer review only - http://bmjopen.bmj.com/site/about/guidelines.xhtml

42. Boaz, A., et al., *How to engage stakeholders in research: design principles to support improvement*. Health research policy and systems, 2018. **16**(1): p. 60.

43. Deverka, P.A., et al., *Stakeholder participation in comparative effectiveness research: defining a framework for effective engagement*. Journal of Comparative Effectiveness Research, 2012. **1**(2): p. 181-194.

44. Mathur, V.N., A.D. Price, and S. Austin, *Conceptualizing stakeholder engagement in the context of sustainability and its assessment*. Construction Management and Economics, 2008. **26**(6): p. 601-609.

For peer review only

For peer review only - http://bmjopen.bmj.com/site/about/guidelines.xhtml

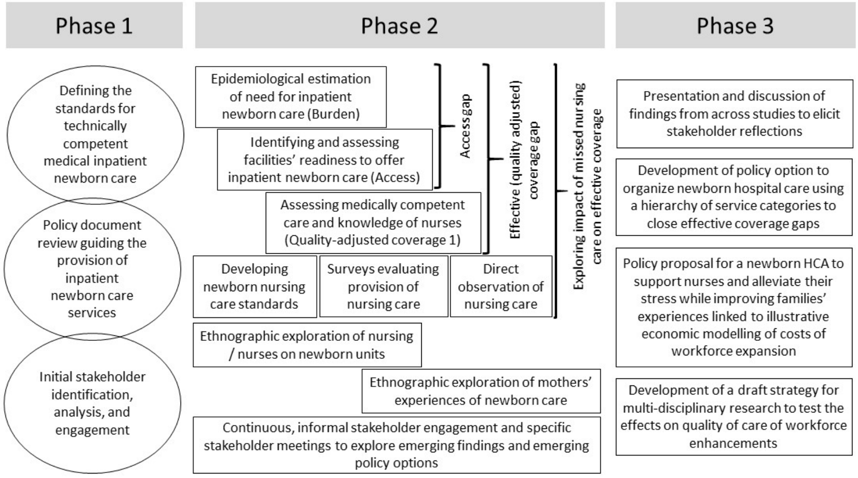

Schematic of HSD-N research components, their inter-relationship and infused stakeholder engagements throughout the research cycle

338x190mm (96 x 96 DPI)

COREQ (COnsolidated criteria for REporting Qualitative research) Checklist

A checklist of items that should be included in reports of qualitative research. You must report the page number in your manuscript where you consider each of the items listed in this checklist. If you have not included this information, either revise your manuscript accordingly before submitting or note N/A.

Topic	Item No.	Guide Questions/Description	Reported on Page No.
**Domain 1: Research team and reflexivity**			
Personal characteristics			
Interviewer/facilitator	1	Which author/s conducted the interview or focus group?	
Credentials	2	What were the researcher's credentials? E.g. PhD, MD	
Occupation	3	What was their occupation at the time of the study?	
Gender	4	Was the researcher male or female?	
Experience and training	5	What experience or training did the researcher have?	
Relationship with participants			
Relationship established	6	Was a relationship established prior to study commencement?	
Participant knowledge of the interviewer	7	What did the participants know about the researcher? e.g. personal goals, reasons for doing the research	
Interviewer characteristics	8	What characteristics were reported about the inter viewer/facilitator? e.g. Bias, assumptions, reasons and interests in the research topic	
**Domain 2: Study design**			
Theoretical framework			
Methodological orientation and Theory	9	What methodological orientation was stated to underpin the study? e.g. grounded theory, discourse analysis, ethnography, phenomenology, content analysis	
Participant selection			
Sampling	10	How were participants selected? e.g. purposive, convenience, consecutive, snowball	
Method of approach	11	How were participants approached? e.g. face-to-face, telephone, mail, email	
Sample size	12	How many participants were in the study?	
Non-participation	13	How many people refused to participate or dropped out? Reasons?	
Setting			
Setting of data collection	14	Where was the data collected? e.g. home, clinic, workplace	
Presence of non-participants	15	Was anyone else present besides the participants and researchers?	
Description of sample	16	What are the important characteristics of the sample? e.g. demographic data, date	
Data collection			
Interview guide	17	Were questions, prompts, guides provided by the authors? Was it pilot tested?	
Repeat interviews	18	Were repeat inter views carried out? If yes, how many?	
Audio/visual recording	19	Did the research use audio or visual recording to collect the data?	
Field notes	20	Were field notes made during and/or after the inter view or focus group?	
Duration	21	What was the duration of the inter views or focus group?	
Data saturation	22	Was data saturation discussed?	
Transcripts returned	23	Were transcripts returned to participants for comment and/or	

For peer review only

Topic	Item No.	Guide Questions/Description	Reported on Page No.
		correction?	
**Domain 3: analysis and findings**			
Data analysis			
Number of data coders	24	How many data coders coded the data?	
Description of the coding tree	25	Did authors provide a description of the coding tree?	
Derivation of themes	26	Were themes identified in advance or derived from the data?	
Software	27	What software, if applicable, was used to manage the data?	
Participant checking	28	Did participants provide feedback on the findings?	
Reporting			
Quotations presented	29	Were participant quotations presented to illustrate the themes/findings? Was each quotation identified? e.g. participant number	
Data and findings consistent	30	Was there consistency between the data presented and the findings?	
Clarity of major themes	31	Were major themes clearly presented in the findings?	
Clarity of minor themes	32	Is there a description of diverse cases or discussion of minor themes?	

Developed from: Tong A, Sainsbury P, Craig J. Consolidated criteria for reporting qualitative research (COREQ): a 32-item checklist for interviews and focus groups. *International Journal for Quality in Health Care*. 2007. Volume 19, Number 6: pp. 349 – 357

Once you have completed this checklist, please save a copy and upload it as part of your submission. DO NOT include this checklist as part of the main manuscript document. It must be uploaded as a separate file.

For peer review only
